# Performance of 3D-Printed Bionic Conch-Like Composite Plate under Low-Velocity Impact

**DOI:** 10.3390/ma15155201

**Published:** 2022-07-27

**Authors:** Mincen Wan, Dayong Hu, Baoqing Pei

**Affiliations:** 1 Department of Aircraft Airworthiness Engineering, School of Transportation Science and Engineering, Beihang University, Beijing 100191, China; wanmincen@buaa.edu.cn; 2 Aircraft/Engine Integrated System Safety Beijing Key Laboratory, Beijing 100191, China; 3 School of Biological Science and Medical Engineering, Beihang University, Beijing 100191, China

**Keywords:** cross-lamellar, bionic, 3D printing, impact resistance, crack propagation, anti-impact

## Abstract

Biological armors can provide an effective protection against predators. In this study, inspired by conch shell, a new type of bionic composite plate Bio-S was fabricated and compared with two previously investigated designs inspired by beetle exoskeleton (Bio-B) and nacre (Bio-N), as well as with an equivalent monolithic plate formed from the same stiff material designed and manufactured by additive manufacturing. Low velocity impact tests using drop tower were conducted to study their impact resistance. Experimental findings indicated that the Bio-S composite had superior impact resistance compared with the other bionic composites and the monolithic plate. Furthermore, the influence of the ply angle on the impact resistance of the Bio-S composite plate was investigated. The (0°/30°/0°/30°) arrangement was able to provide the highest impact resistance. Finally, the crack propagation mode in Bio-S composites plates was analyzed, enhancing our understanding of the underlying mechanisms during impact. Such findings may lead to the development of superior lightweight protective structures with improved anti-impact performance.

## 1. Introduction

Through millions of years of natural selection and evolution, many organisms have evolved robust protective structures to improve survivability in the surrounding environments [1,2,3]. Various forms of biological exoskeletons or armors, from the hard shells of mollusks to the osteoderms of crocodile [4,5], can perform a critical role in providing effective mechanical protection from predator damage, such as penetration, drilling, crushing, peeling, chipping, hammering, and kinetic strike. Taking inspiration from nature, these unique protective mechanisms have inspired researchers and engineers to develop novel bio-inspired materials and structures with excellent impact resistance and energy absorption (EA) capabilities for aerospace, defense, and transportation applications [1,2,4,5,6,7,8,9,10].

Many innovative structures have been inspired by nature, including bio-inspired flexible scaled armor [11], bio-inspired modular structures [12,13], bionic stab-resistant body armor [14], and bio-inspired sandwich structures [15,16,17,18]. For example, inspired by crocodile armor, Guo et al. [14] designed a stab-resistant substrate with a triangular pyramidal structure, and investigated the stab-resistance behavior and dynamic response mechanisms using numerical simulation and experimental testing of a knife impacting the substrate. Their results revealed that the triangular pyramidal structure generated twice the internal energy of the knife as the flat substrate, which provided a reference for future design and fabrication of stab-resistant clothing. González-Albuixech et al. [12] used the finite element approach to assess the ballistic performances of bio-inspired modular ceramic armors inspired by ganoid fish scales, placoid fish scales, and armadillo osteoderm. According to their findings, the bio-inspired modular armors exhibited more localized damage and crack arrest properties against numerous shots than monolithic constructions. Inspired by the tessellated organization of chiton scales, Connors et al. [11] designed a synthetic flexible scaled armor structure through parametric computational modeling and multi-material 3D printing. The 3D-printed chiton scale assemblies showed strain stiffening behaviors due to the scale interlocking and jamming, which can improve protection by stiffening the scale array, resisting penetration, and maintaining structural integrity. Similar protection mechanisms were also found in fish scales. Liu et al. [19] developed a bio-inspired composite scale-like protection system. It was found that the area density of the bio-inspired composite scale can be reduced by 12.5% while maintaining the same ballistic performance at the impact velocity of 878 m/s compared with pure silicon carbide scales. This bio-inspired composite scale system can be regarded as suitable for developing a novel bionic protective armor. The fibrous layered helicoidal structure found in the forelimb of mantis shrimp can maintain structural integrity under high intense loads, dissipate impact energy, and reduce the thickness of damage to improve residual strength [20,21,22]. Wu et al. [23] proposed an architecture that combined Bouligand and nacreous staggered structures, inspired by the survival war between the ‘spear’ of mantis shrimps with Bouligand-type microstructures and the ‘shield’ of abalone with nacreous staggered architectures. This structure exhibited excellent fracture resistance with crack orientation insensitivity.

Moreover, the cross-lamellar structures existing in nacre [24] and conch [25] have also attracted great attention because it can provide high strength and superior fracture toughness through well-known fracture mechanisms: crack deflection and bridging, which have been comprehensively reviewed in Refs [1,5,26,27]. Recently, the 3D printing technique was used to mimic nacre-like and conch-like structures. Tran et al. [28] used the Fused Deposition Modeling (FDM) 3D printing technique to successfully mimic a nacre-like structure, in which Acrylonitrile butadiene styrene (ABS) was used as the stiff material to mimic the nacre-like tablets, and poly lactic acid (PLA) or thermoplastic polyurethane (TPU) was used to mimic the soft matrix. Wei et al. [29] designed a novel nacre-like structure with a gradient of cell size. The results of pendulum impact experiments and finite element analysis indicated that the gradient change in the cell size can improve the stress and strain distribution, particularly increasing the strain energy density at the impact region. Moreover, increasing the cell size gradient can also effectively increase the total strain energy stored in the gradient structure and significantly improve the impact resistance of the nacre-like structure. Salinas et al. [30] developed third-order lamellar conch-like structures through 3D printing, and indentation tests were conducted to investigate the influence of layer orientations on their mechanical properties. The results demonstrated the alignment angles of ±45° were reported to provide the highest resistance to penetration. Gu et al. [31,32] printed conch-like plates using VeroMagenta and TangoBlackPlus as stiff and soft materials, respectively. Drop tower experiments were carried out to demonstrate the benefits of the conch-like plate over an equivalent monolithic plate formed from the same stiff material. Results showed the conch-like plates had a prominent increase in fracture toughness compared with the monolithic panel. Liu et al. [33] used the 3D printing technique to fabricate the cross-laminated hierarchical structural of the bionic S. gigas shell with different cross-angles (0°, 20°, 40°, 60°, and 90°), and studied their strengthen and toughen mechanisms. It was found that a suitable cross-angle can significantly regulate the stress distribution in the hard and soft phases of the hierarchical structure, improving the bearing capacity and EA. Zhang et al. [10] fabricated three types of Mg-Ti composites with bioinspired nacre-like, Bouligand, and conch-like architectures through pressureless infiltration of pure Mg melt into 3D printed Ti-6Al-4V scaffolds. By comparing their mechanical characteristics, the conch-like structure proved to be the most effective among the three bioinspired architectures in strengthening materials, delocalizing damage, and resisting crack propagation. Moreover, it had the best combination of mechanical properties, including strength, elongation, fracture work, fracture toughness, and impact toughness. As mentioned above, it can be concluded that the bionic methods are able to improve the impact resistance performance, thus providing more inspiration from bionic design to develop a novel protective structure.

From the literature review, previous studies have mainly focused on the material composition, crack propagation, failure mechanism, and bionic preparation. However, there are few studies on the impact resistance characteristics of the representative cross-lamellar structure. Additionally, the interior structure of the natural conch shell was fixed and could not be changed, which brought great limitations to explore the influence of the structural parameters, such as ply angle, on the impact resistance. To address this issue, inspired by conch shell, we designed a bionic conch-like composite plate. In addition, since nacre shell and beetle exoskeleton featured similar stiff–soft phase combination configurations to the conch shell, previously developed bionic nacre-like and beetle exoskeleton composite plates [30] were also used to compare with the bionic conch-like composite plate. An equivalent monolithic plate was also used to compare with these three bionic composite plates. These composite plates with different architectures were fabricated by the multi-material 3D printing technique. Then drop tower tests at different impact energy levels were carried to investigate the low-velocity impact damage behaviors and energy dissipation capability of these bionic composite plates. The influence of the stacked configuration on the impact resistance as well as the crack propagation was also discussed.

## 2. Materials and Methods

### 2.1. Bionic Composites Design and Fabrication

**Figure 1:**
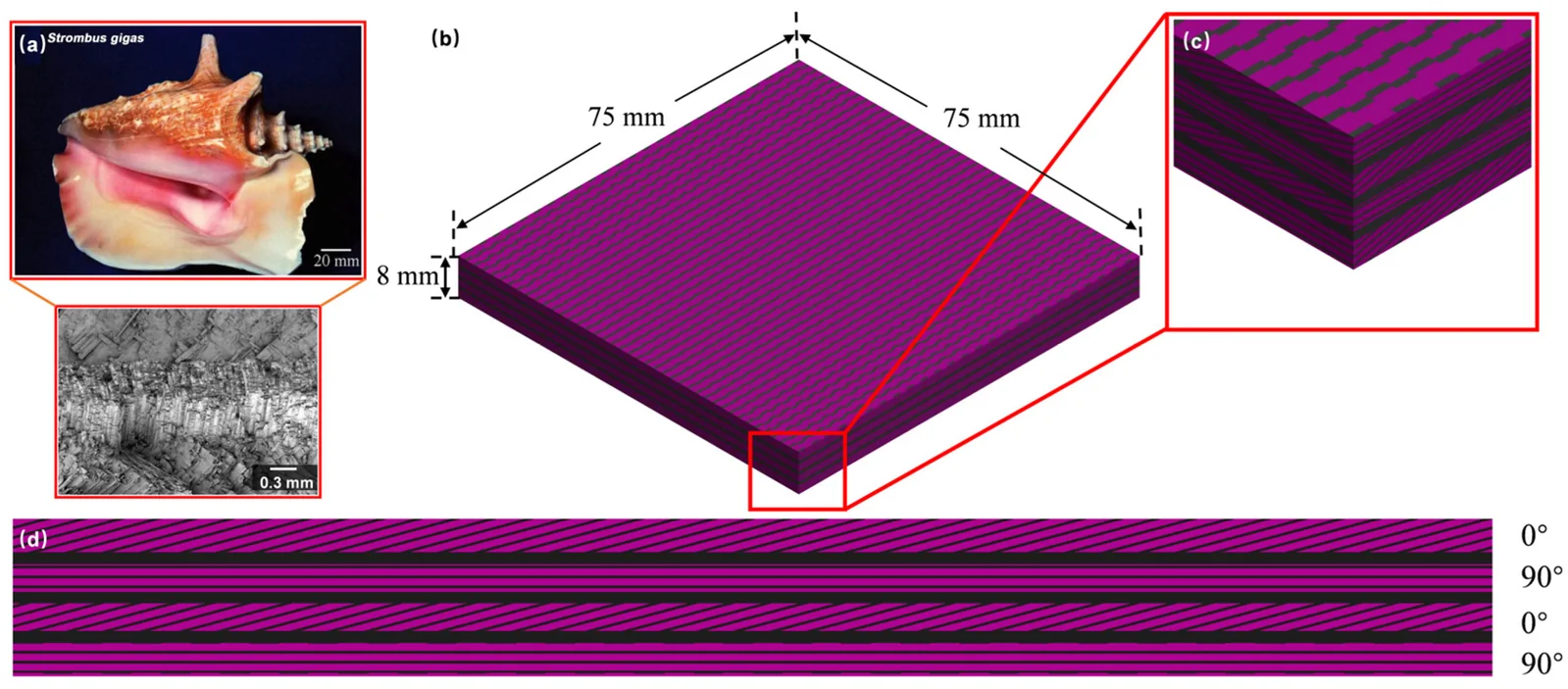
Schematic diagram of Bio-S inspired by mollusk shell [32] (**a**) microstructure of conch shell (Strombus gigas); (**b**) schematic diagram of Bio-S specimen model; (**c**) local feature of bio-S structure; (**d**) the side view of the Bio-S structure.

**Figure 2:**
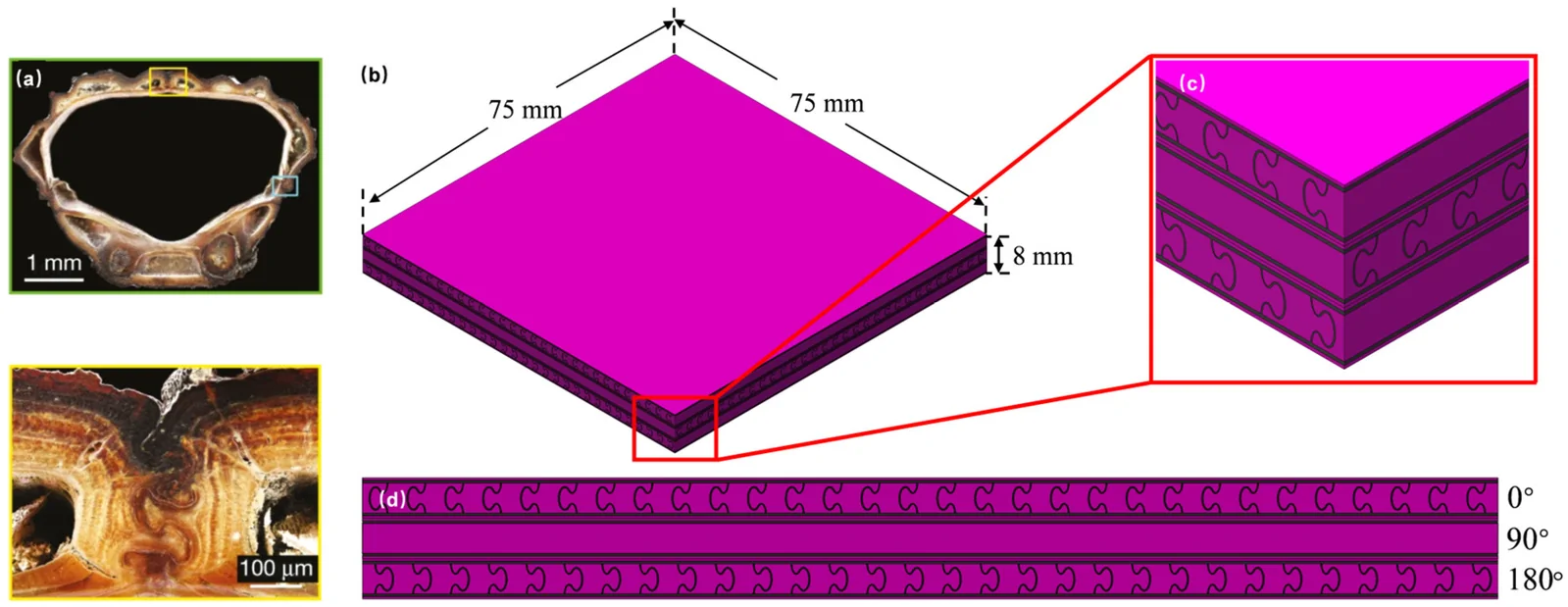
Schematic diagram of Bio-B inspired by beetle exoskeleton [34] (**a**) microstructure of beetle exoskeleton; (**b**) schematic diagram of Bio-B specimen model; (**c**) local feature of bio-B structure; (**d**) the side view of the Bio-B structure.

**Figure 3:**
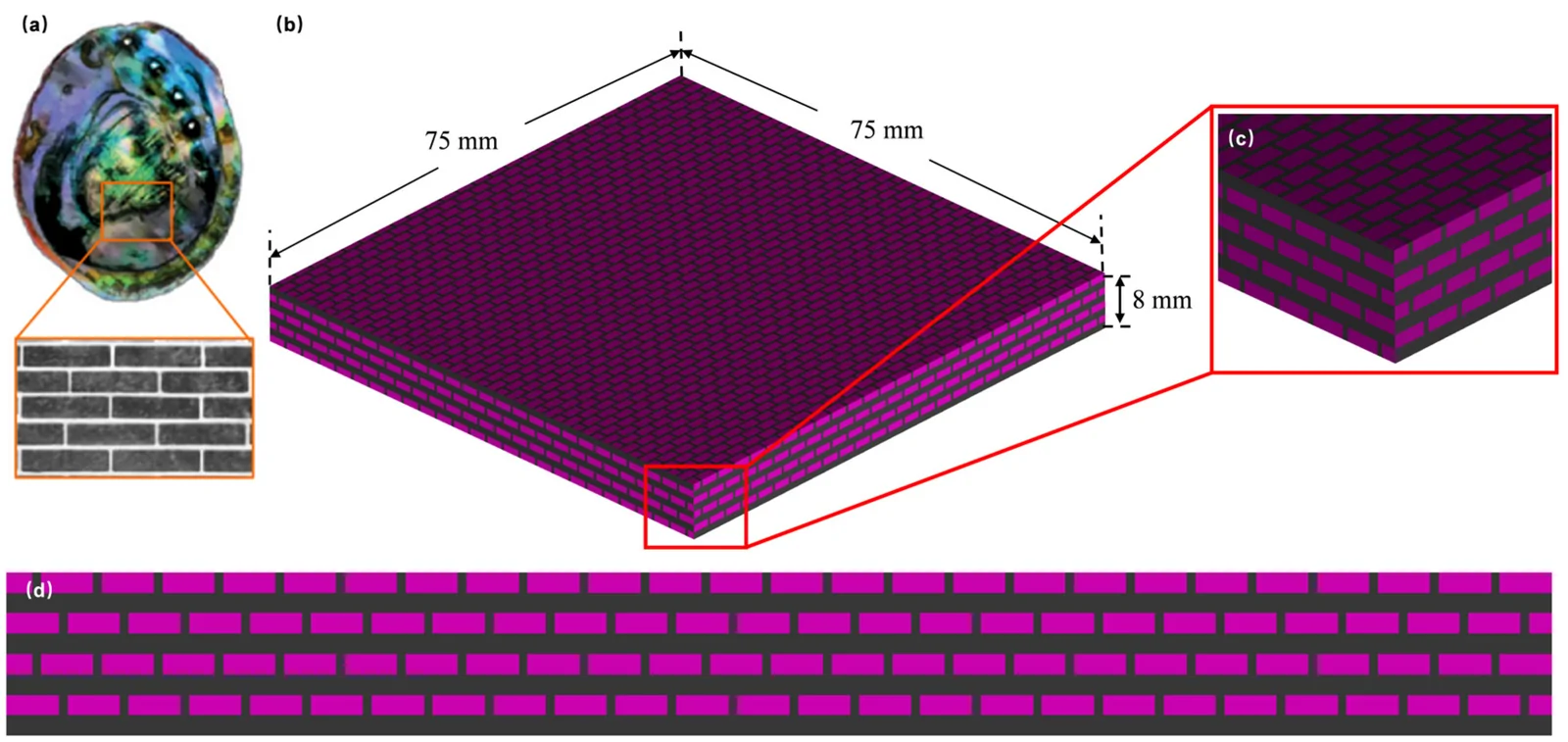
Schematic diagram of Bio-N inspired by nacre [31] (**a**) microstructure of nacre; (**b**) schematic diagram of Bio-N specimen model; (**c**) local feature of bio-N structure; (**d**) the side view of the Bio-N structure.

Inspired by the Strombus gigas conch shell, beetle exoskeleton, and nacre, three types of bionic composite plates named Bio-S, Bio-B, and Bio-N were designed, respectively. Figure 1, Figure 2 and Figure 3 demonstrate their detailed geometric models. Each bionic composite plate included two phases: a relatively stiff phase ‘purple color’ and a relatively compliant phase ‘black color’. The volume fractions of the stiff phase were 55.3%, 82.1%, and 64.7% for Bio-S, Bio-B, and Bio-N, respectively. It can be noted that these three bio-composite plates had different volume fraction. If we instead kept the same volume fraction of stiff phase, it would bring difficulties for geometric modeling and make the design inconsistent with the actual biological structure [32]. The same problem was also reported by Gu et al. [32]. All samples were kept at the same mass of 52 g.

**Figure 4:**
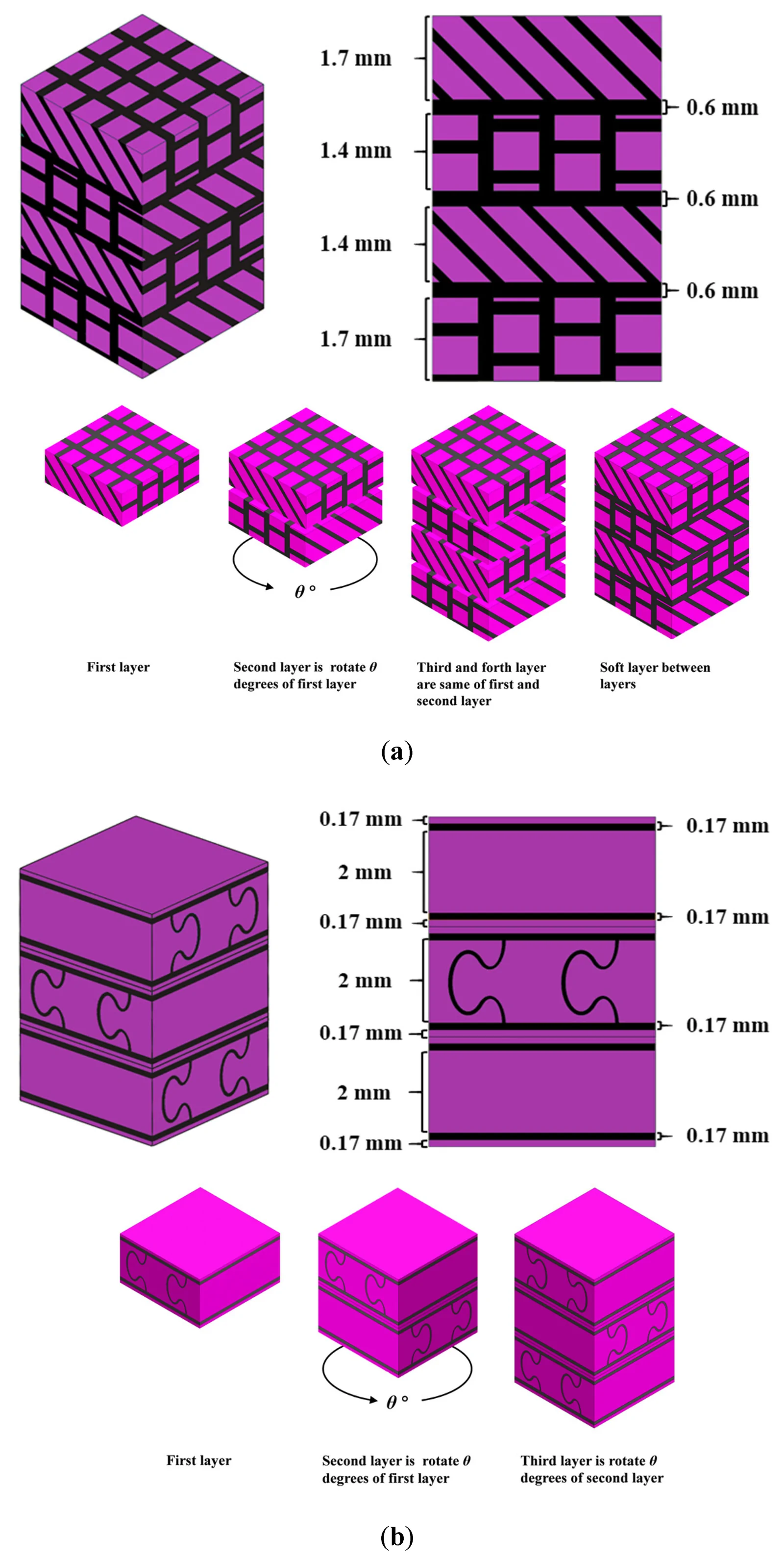

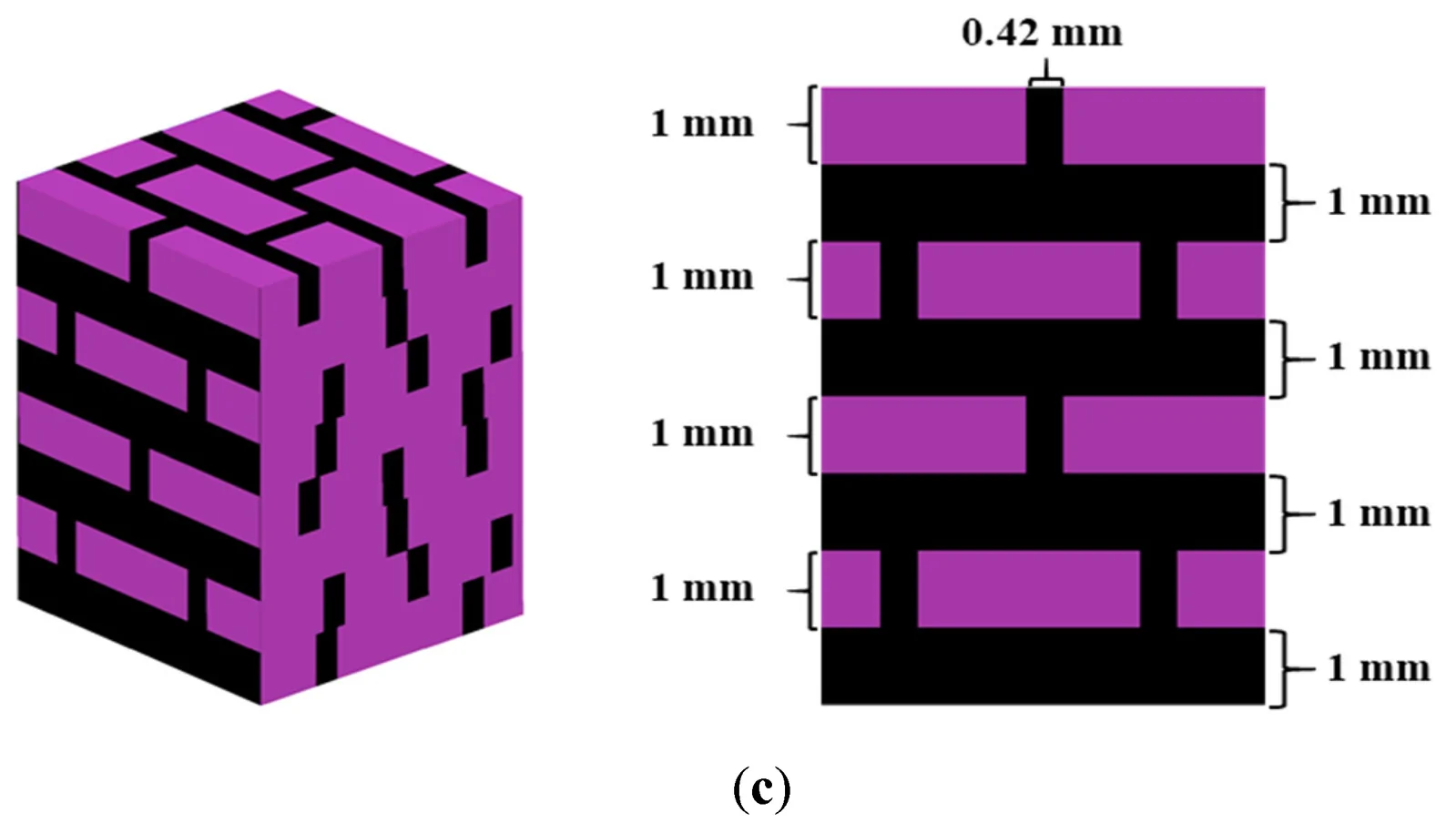
Schematic diagram of typical unit cell: (**a**) Bio-S; (**b**) Bio-B; (**c**) Bio-N.

The Bio-S (Figure 1) was composed of four plies and assembled following the sequence: (0°/90°/0°/90°), based on the hierarchical structure configuration reported by Gu et al. [32]. Figure 4a showed the dimensions of the unit cell. The second level hierarchy structure was adopted as shown in Figure 4a, as the impact performance of the bionic conch shell structure by adding the second level hierarchy was 70% higher than that of the single level hierarchy [32]. Each layer was composed of several parallel 1st-order lamellae. The 1st-order lamellae were a lamella composed of unidirectional layers of lath-like 2nd-order lamellae, and the orientation of the second level hierarchy in two adjacent 1st-order lamellae was ±45° alternately. To create the laminate of Bio-S, a multilayer unit cell was formed by rotating single unit cell (see Figure 1) and repeated in the in-plane direction. Here, the multilayer unit cell was repeated more than 15 times in each in-plane direction to eliminate the size effects and better capture the global anti-impact performance [32]. The Bio-B (Figure 2) was a composite plate, characterized with the tenon inserted in the mortise, as reported by Rivera et al. [34] and Zhang et al. [35]. Figure 4b showed the typical unit cell of Bio-B, where the 0° layer was designed in the direction of 0° section perpendicular to the suture line of carapace, rotated 90° clockwise for 90° layer, and rotated 180° clockwise for 180° layer. The Bio-N (Figure 3) was a typical brick–mortar configuration [31], and its unit cell can be seen in Figure 4c. Additionally, a monolithic plate (MSP) formed from the same stiff phase was also created to compare with the bionic composite plates. All designs were fabricated using multi-material 3D-printing.

**Figure 5:**
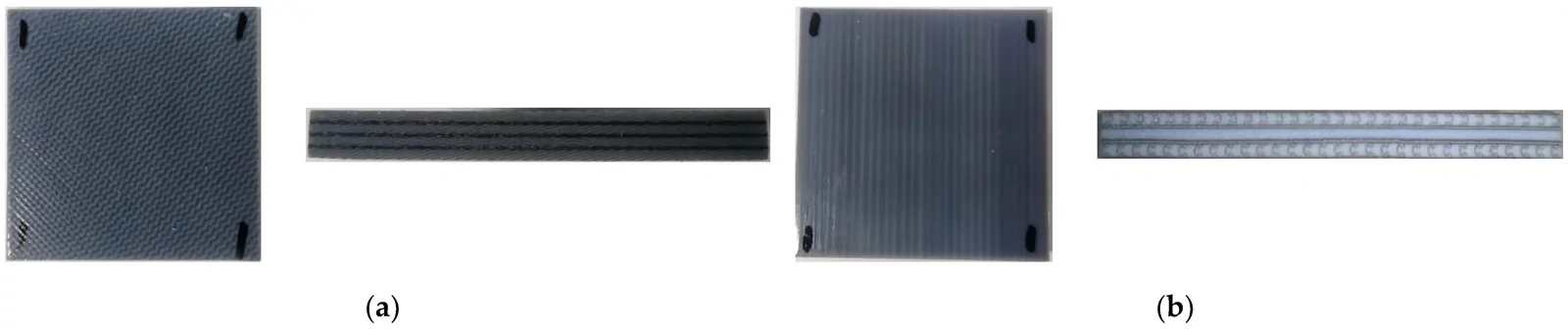

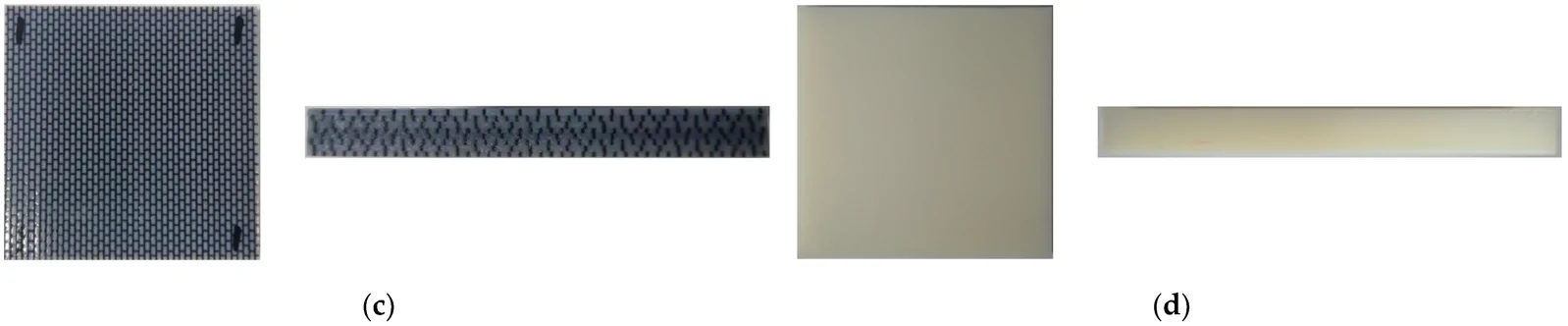
Specimen of each structure: (**a**) Bio-S [0°/90°/0°/90°]; (**b**) Bio-B [0°/90°/180°], (**c**) Bio-N and (**d**) MSP. Reprinted with permission from Ref. [36]. Copyright 2022 Elsevier Ltd.

A Stratasys Objet 260 3D printer was used to fabricate various samples, which can simultaneously print multiple distinct materials with a near-perfect interface. The 3D printer had a resolution of 16 μm, and it used a multi-material PolyJet technology with dual materials, allowing precise fabrication of the bionic composite plates with the soft-and-stiff phases. The photosensitive resin materials were ejected through printing heads onto a printing tray. An ultraviolet lamp trailed the printing heads, and immediately cured each layer of resin material after it was extruded. Due to this instantaneous in situ curing process, excellent adhesion could be achieved between similar and dissimilar materials [32]. The commercial geometric modeling software SolidWorks was used to establish the structural geometry model, and then exported the STL file to the software Objet Studio to set the printing parameters. As shown in Figure 1, Figure 2 and Figure 3, the verowhite was utilized as the stiff material marked by the purple color, and the tangoblackplus was used as soft material marked by the black color. The verowhite and tangoblackplus were Stratasys proprietary photopolymers. To print the verowhite and tangoblackplus properly, the temperature of the print head was set to 68~71 °C during printing and the temperature of the printing space was set to 18~25 °C. Figure 5 showed the printed specimens named Bio-S, Bio-B, Bio-N, and MSP with the dimensions: 75 mm (length) × 75 mm (width) × 8 mm (height), respectively.

**Figure 6:**
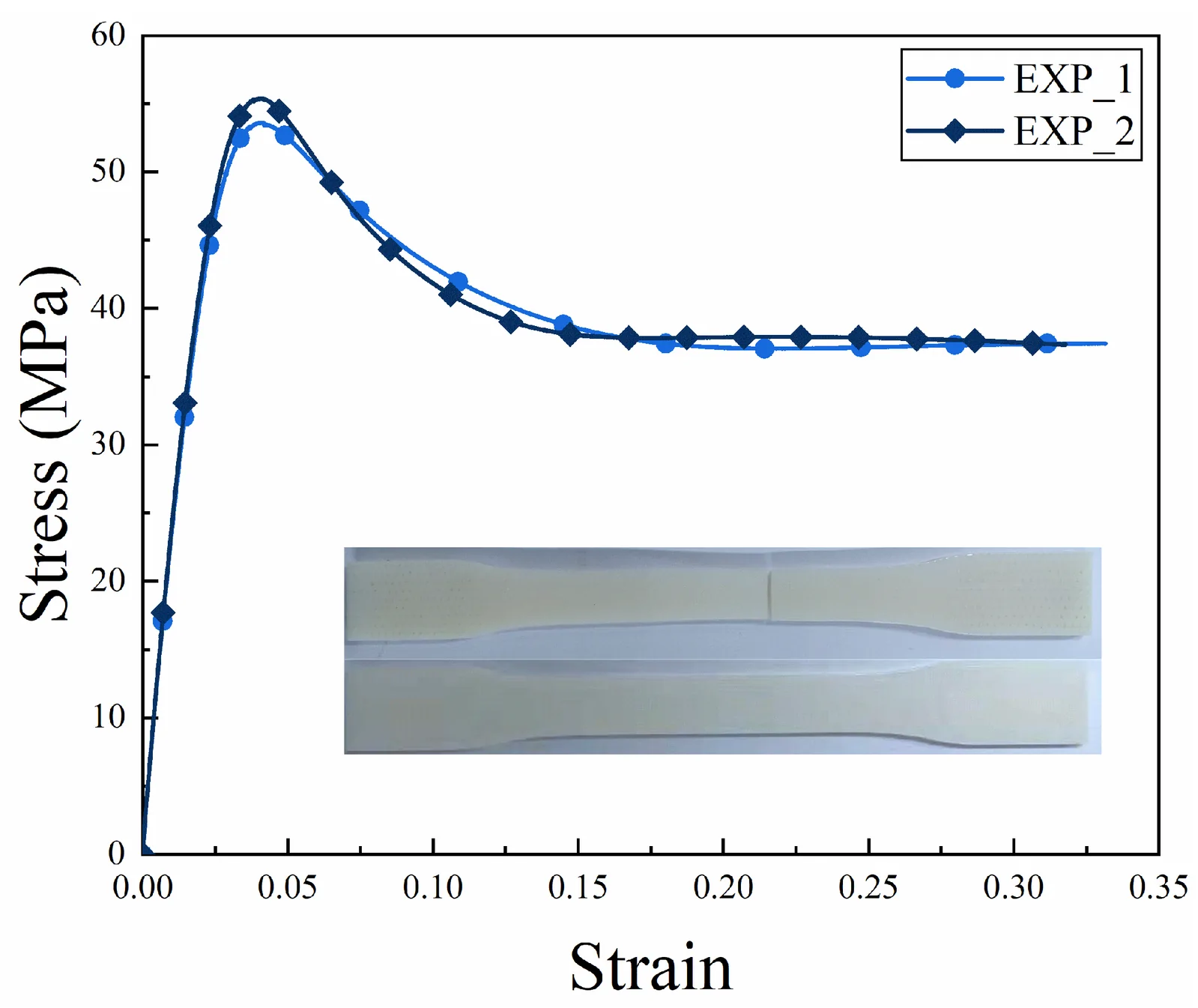
Tensile stress-strain curve of verowhite.

The quasi-static mechanical properties of the stiff material were measured using an Instron machine according to ASTM D412. The stress-strain curve of the stiff material obtained from the uniaxial tensile tests was presented in Figure 6, and the basic mechanical properties of stiff materials were listed in Table 1. For the soft material, the mechanical properties were the same as Gu et al. [32]. The soft material was the same as Gu et al. [32], and its mechanical properties were as follows: the density of 1200 kg/m^3^, bulk modulus of 10 GPa, shear modulus of 0.83 MPa, and elongation of 1.4.

### 2.2. Drop Tower Test

**Figure 7:**
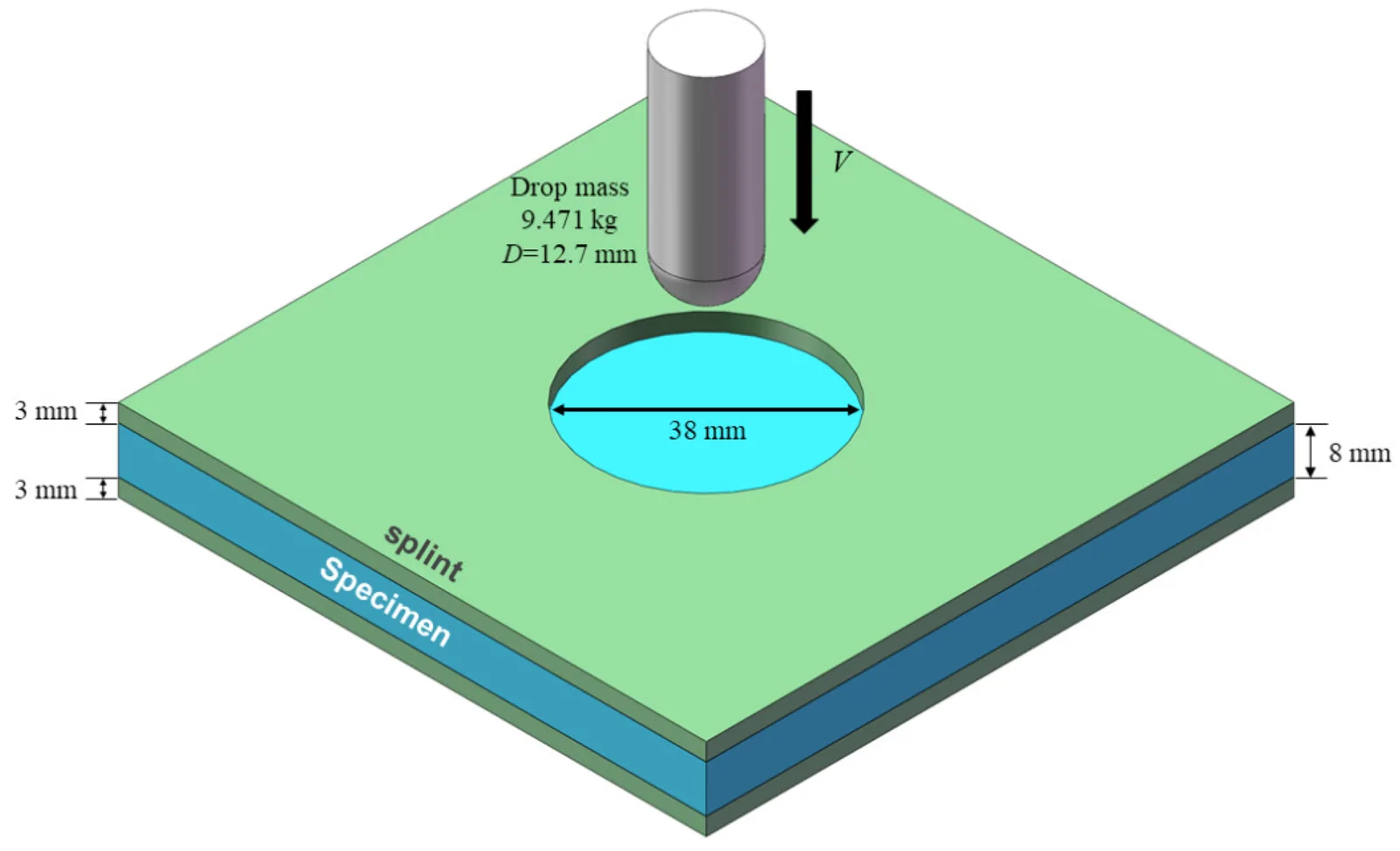
Schematic diagram of the drop tower test.

To investigate the low-velocity impact resistance characteristics of Bio-S, Bio-B, Bio-N, and MSP, drop tower tests were conducted using an Instron Ceast 9350 drop tower instrument with an impactor mass of 9.471 kg, as shown in Figure 7. According to the findings of Gu et al. [32], the critical impact energy for verowhite (stiff material) should be less than 15 J. As a result, the energy of 15 J was selected as the lowest impact energy level in the drop tower tests. To assess the impact resistance of the samples under different impact velocities, three impact energy levels of 15 J, 20 J, and 25 J were selected, corresponding to impact velocities of 1.78 m/s, 2.06 m/s, and 2.31 m/s, respectively. The different impact energies can be obtained by adjusting impactor drop heights. The impactor had a hemispherical nose with 12.7 mm diameter, while each specimen was sandwiched between two splints with a 38 mm diameter central hole before the impactor free fell onto the specimen. The load-time curves of the experiment were recorded by the Das 64K sensor with a sampling frequency of 5000 Hz.

## 3. Results and Discussion

### 3.1. Impact Resistances of Bio-S, Bio-B, Bio-S and MSP

**Figure 8:**
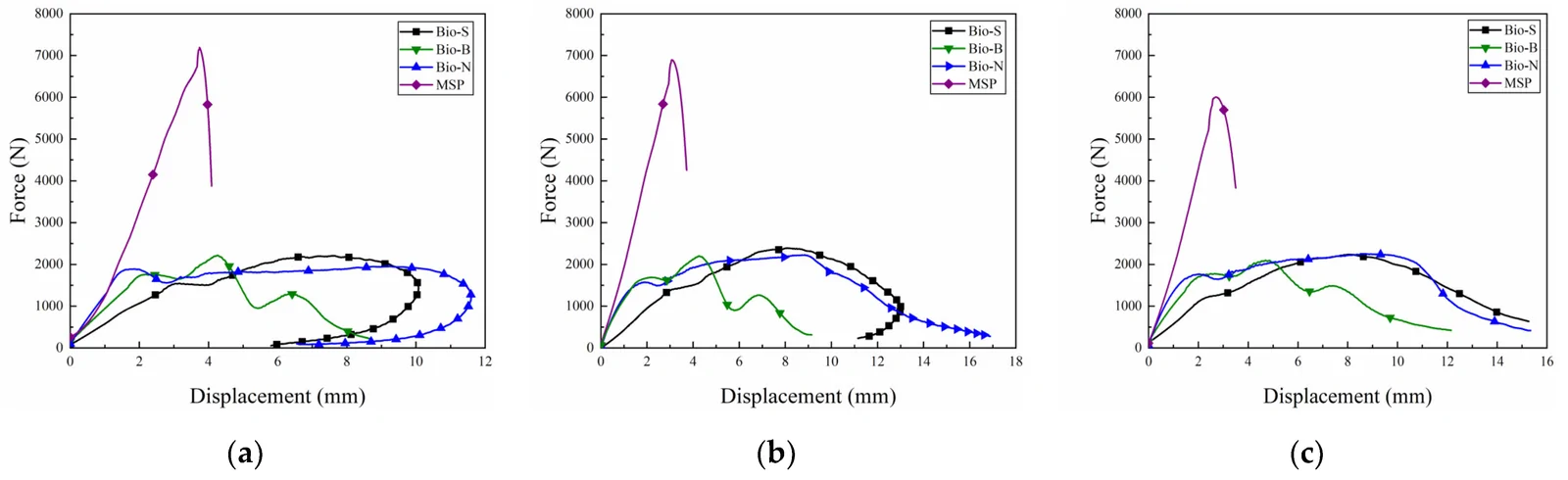
Comparison of force–displacement curves between Bio-S and the other three composite plates [36] under different impact energies: (**a**) 15 J; (**b**) 20 J; (**c**) 25 J.

**Figure 9:**
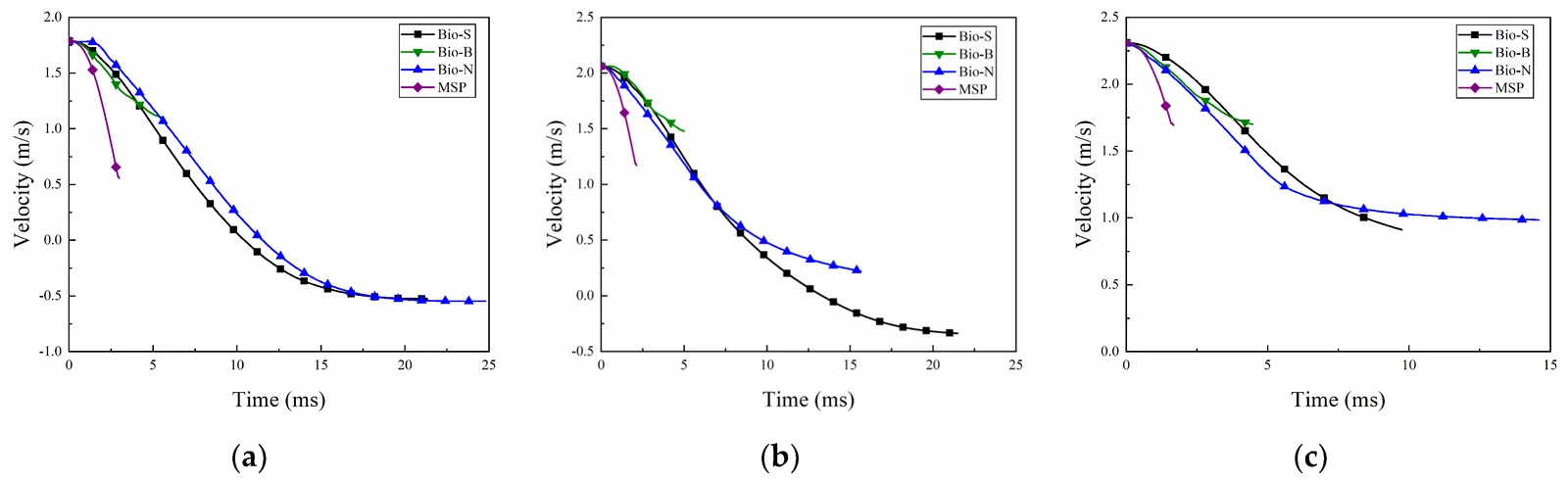
Comparison of velocity-time curves between Bio-S and the other three composite plates [36] under different impact energy levels: (**a**) 15 J; (**b**) 20 J; (**c**) 25 J.

**Figure 10:**
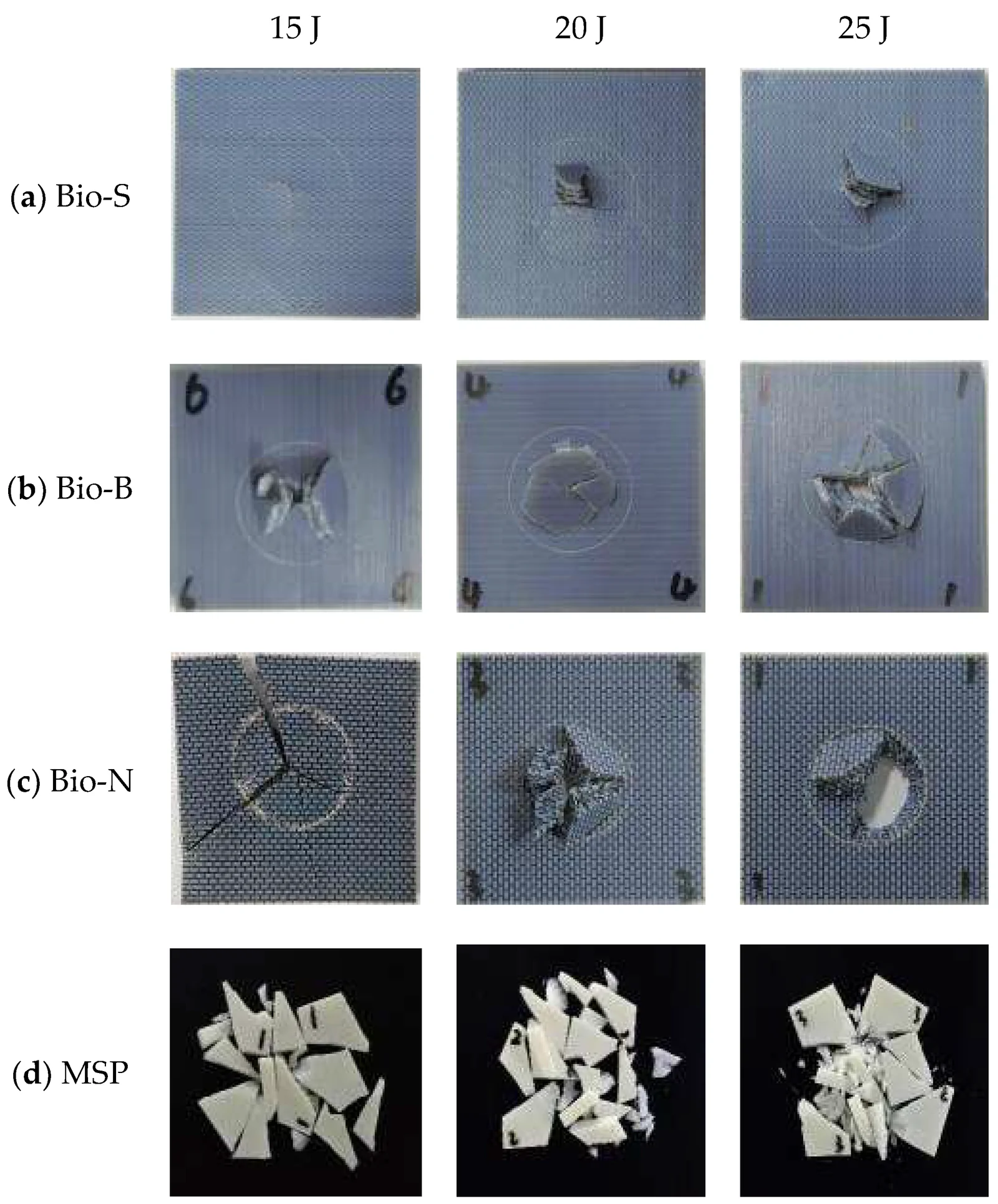
The damage patterns of Bio-S and the other composite plates. Reprinted with permission from Ref. [36]. Copyright 2022 Elsevier Ltd.

The force-displacement curves and velocity-time history curves of Bio-S, Bio-B, Bio-N, and MSP obtained from the drop tower tests under different impact energies were presented in Figure 8 and Figure 9, where 15 J, 20 J, 25 J represented different impact energy levels. The damage morphologies of all specimens were also shown in Figure 10. From Figure 8, the force-displacement curves of MSP under the three impact energy levels showed the similar shape, characterized by that the impact force increasing rapidly and then dropping suddenly after reaching the peak force. In Figure 10, it can be observed that all MSP specimens broke into many pieces under different impact energies, while large residual velocities can be seen in Figure 9. Based on the test results of MSP from Figure 8, Figure 9 and Figure 10, it can be concluded that MSP failed in a brittle manner immediately under the impact force, indicating a low impact resistance. For Bio-B, the shape of each force-displacement curve in Figure 8 has three peaks and troughs. When the impactor contacted the Bio-B specimen, the force began to increase almost linearly, and then the force reached the first peak and the second peak successively when the crack initiation occurred. After the second peak force, the force dropped gradually to zero. From Figure 10, it can be observed that the three Bio-B specimens locally fragmented at all impact energies. In contrast, the other two bionic composite plates: Bio-S and Bio-N showed better impact resistance. It can be observed from Figure 8a–c that force-displacement curves for each specimen can be divided into two evident stages. In the first stage, the impact force increased with one small fluctuation and then gradually reached the peak force, corresponding to crack initiation. In the second stage, the force-displacement curves of Bio-N and Bio-S specimens under 15 J impact energy displayed similar elastic recovery tendency (Figure 8a), indicating that they could resist the crack propagation leading to crack holes. Moreover, the force-displacement curves of these specimens under impact energy level 25 J also exhibited a similar gradual decrease to zero behavior with no elastic recovery (Figure 8c), suggesting they failed to resist the penetration (see 25 J in Figure 10a,c). However, under the impact energy level of 20 J, there was an evident difference between the shape of force-displacement curves of Bio-S and Bio-N. Only Bio-S showed an elastic recovery behavior. The reason can be explained based on the damage patterns in Figure 10, which showed that the impactor completely went through Bio-N, but failed to go through Bio-S under the impact energy of 20 J.

From Figure 8, it can be observed that MSP had the highest peak force and the shortest displacement, while Bio-B had a lower impact force but a slightly longer displacement than MSP. The abrupt drop in impact force indicated that MSP was quickly destroyed, while the relatively short displacement of MSP and Bio-B suggested that they had lower energy dissipation capability than the other two bionic composite plates. Furthermore, as shown in Figure 9, it can be clearly found that there are still large residual velocities for MSP and Bio-B under different impact energy levels, while the damage morphologies also revealed that even at the lowest velocity, MSP and Bio-B, were not able to prevent impactor crack propagation leading to crack holes, as shown in Figure 10. Accordingly, it can be considered that MSP and Bio-B exhibited the worst impact resistance. In contrast, as seen from the curves in Figure 8, Bio-S and Bio-N showed a relatively higher force and longer displacement, leading to more energy dissipation. It can be observed from Figure 9 that the velocities of Bio-S and Bio-N rapidly decreased to or close to 0 m/s, which also indicated most of the impact energy could be dissipated. The velocity-time curves shown in Figure 9a revealed that Bio-S and Bio-N could resist the impact energy level of 15 J without crack propagation leading to crack holes, and the impactor rebounded. However, from Figure 9b, Bio-S could fully dissipate the initial kinetic energy, but Bio-N could not. In addition, from Figure 9c, the residual velocities of Bio-N were higher than Bio-S, although the initial kinetic energies were not completely dissipated by Bio-N and Bio-S. By comparing the deformation patterns as shown in Figure 10, it can be concluded that under the lowest impact energy of 15 J, significant fracture occurred in Bio-N, while only a few cracks were visible in Bio-S. The above analysis and observations indicate that Bio-N had lower impact resistance than that of Bio-S.

Furthermore, EA can be calculated by the integral of the force-displacement curve in Figure 8. Velocity change ΔV was the difference between initial velocity and final velocity in Figure 9. EA, peak force, and ΔV, as good indicators to directly compare the impact resistance, were summarized in Table 2. It can be observed from Table 2 that EA, ΔV, and peak force of Bio-S had an obvious tendency to be higher than those of Bio-N. In terms of EA, Bio-S was 7.8% higher than Bio-N under the 25 J impact energy level. In terms of ΔV, Bio-S was 10.2% and 13.4% higher than Bio-N under impact energy levels of 20 J and 25 J, respectively. From the above experimental results, it can be concluded that Bio-S showed the best impact resistance, Bio-N showed the second-best impact resistance, and MSP and Bio-B showed the worst impact resistance.

### 3.2. Effect of the Ply Angle on the Impact Resistance of Bio-S

**Figure 11:**
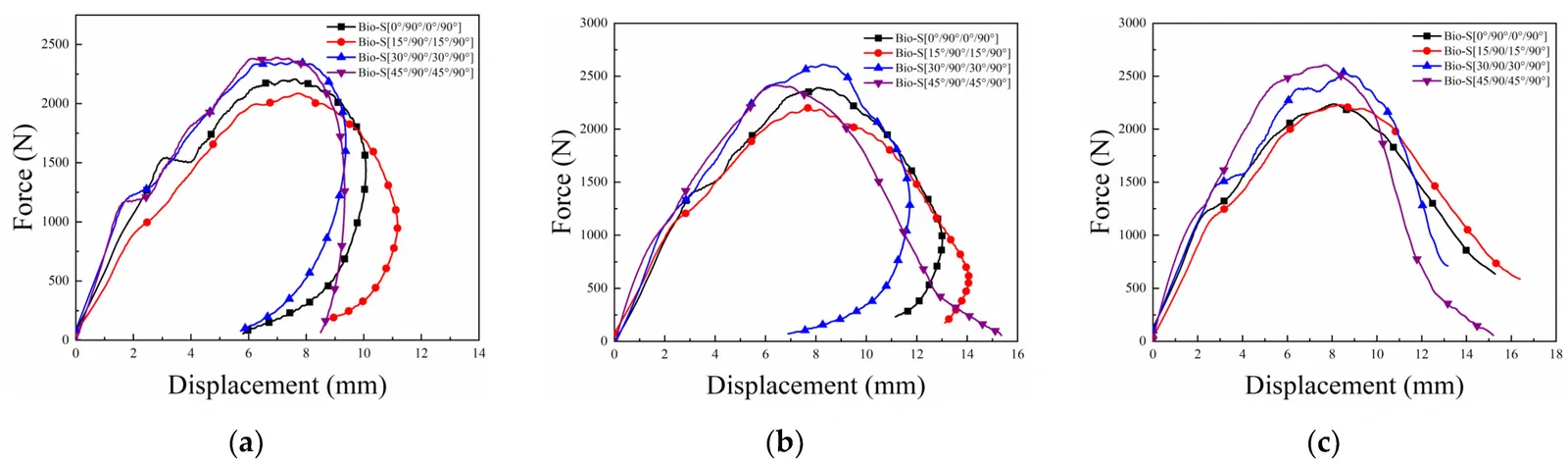
Force-displacement curves of the Bio-S composite plates with different angles of first-third layers configurations under different impact energy levels: (**a**) 15 J; (**b**) 20 J; (**c**) 25 J.

Compared with MSP, Bio-B, and Bio-N in Section 3.1, Bio-S showed the best impact resistance. To further investigate the influence of ply angle on the impact resistance of Bio-S, two groups of specimens were established. The first group fixed the ply angles of the second and fourth layers, and changed the ply angles of the first and third layers, which were (0°/90°/0°/90°), (15°/90°/15°/90°), (30°/90°/30°/90°), and (45°/90°/45°/90°) configurations. The second group varied the angle of the second and fourth layers, which included (0°/30°/0°/30°), (0°/60°/0°/60°), and (0°/90°/0°/90°). The (30°/90°/30°/90°) and (0°/60°/0°/60°) had the same configuration. The structure of different lamination angles is shown in Figure 11.

**Figure 12:**
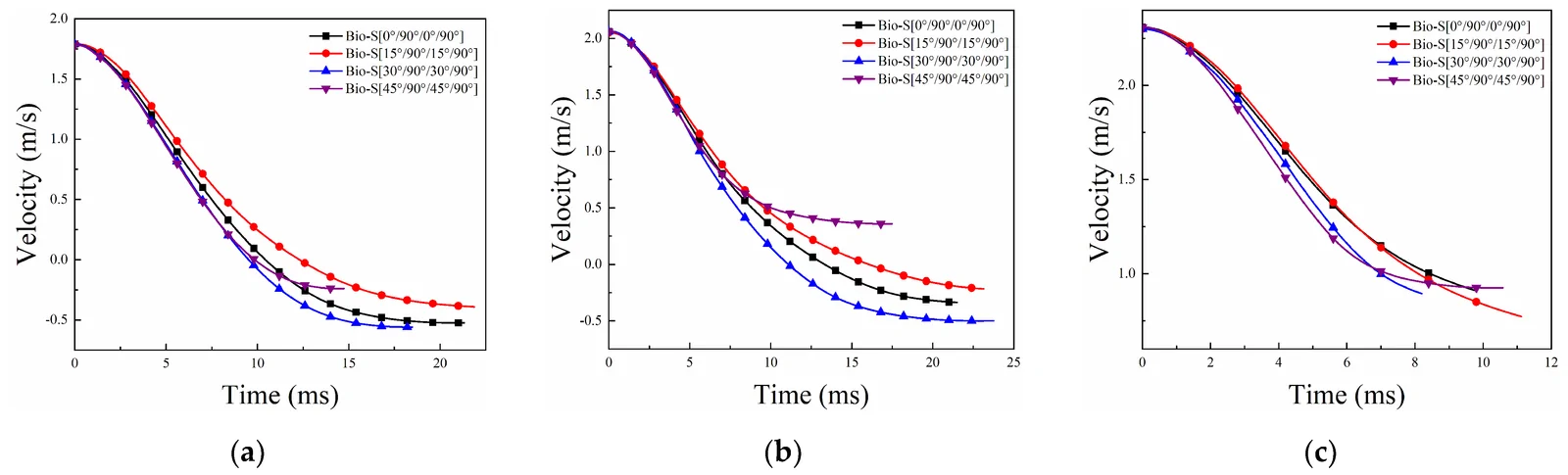
Velocity-time curves of the Bio-S composite plates with different angles of first-third layers configurations under different impact energy levels: (**a**) 15 J; (**b**) 20 J; (**c**) 25 J.

**Figure 13:**
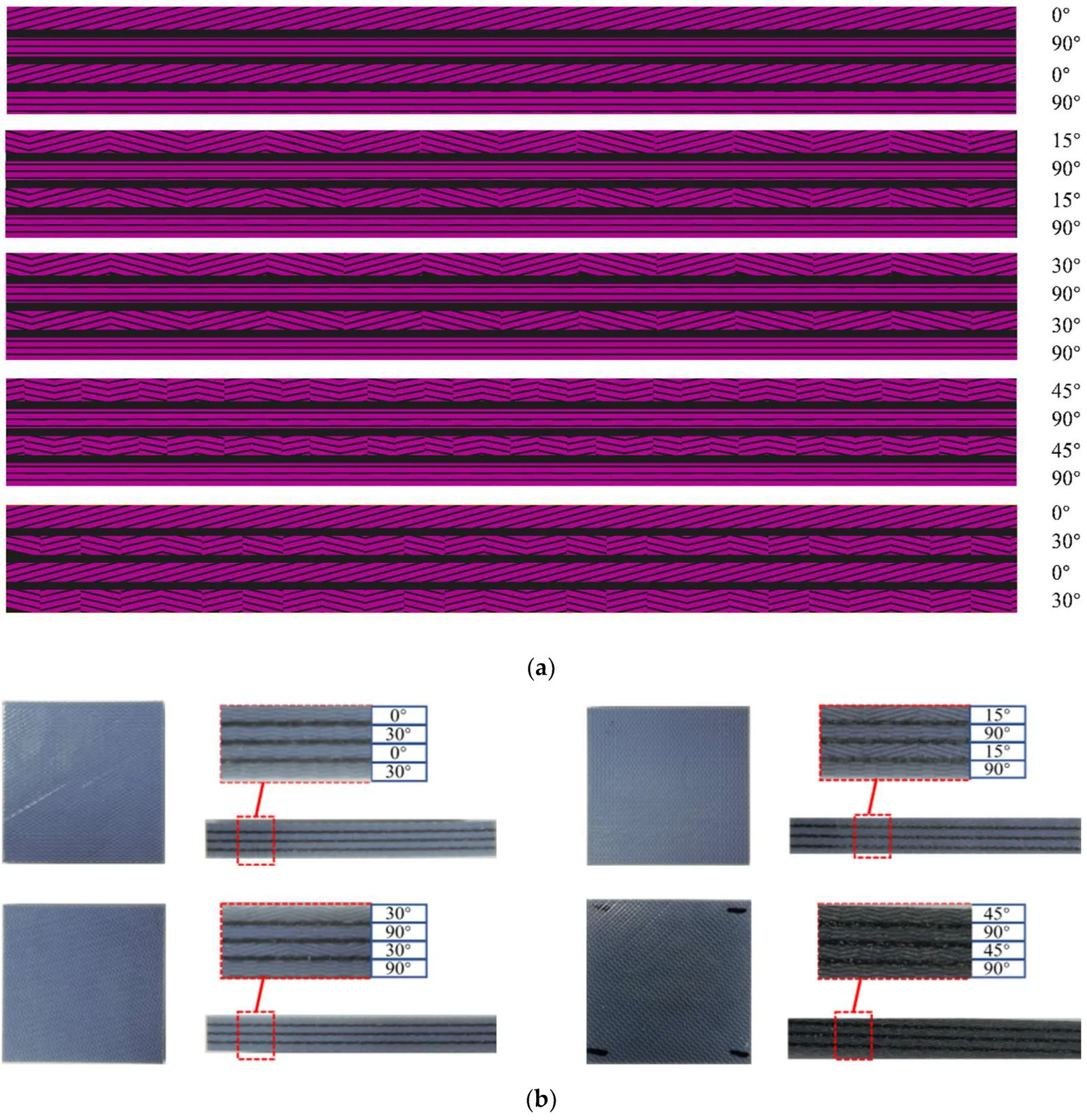
(**a**) Schematic diagram of different layers of Bio-S; (**b**) the corresponding test specimens.

**Figure 14:**
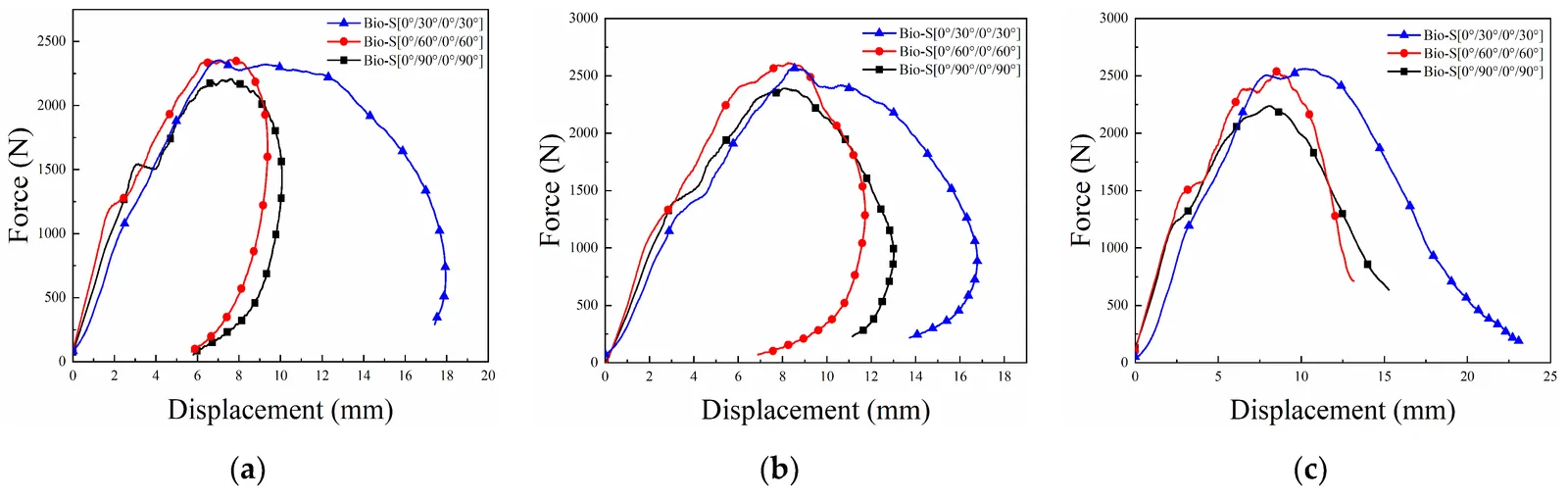
Force-displacement curves of the Bio-S composite plates with different angles of middle layer configurations under different impact energy levels: (**a**) 15 J; (**b**) 20 J; (**c**) 25 J.

**Figure 15:**
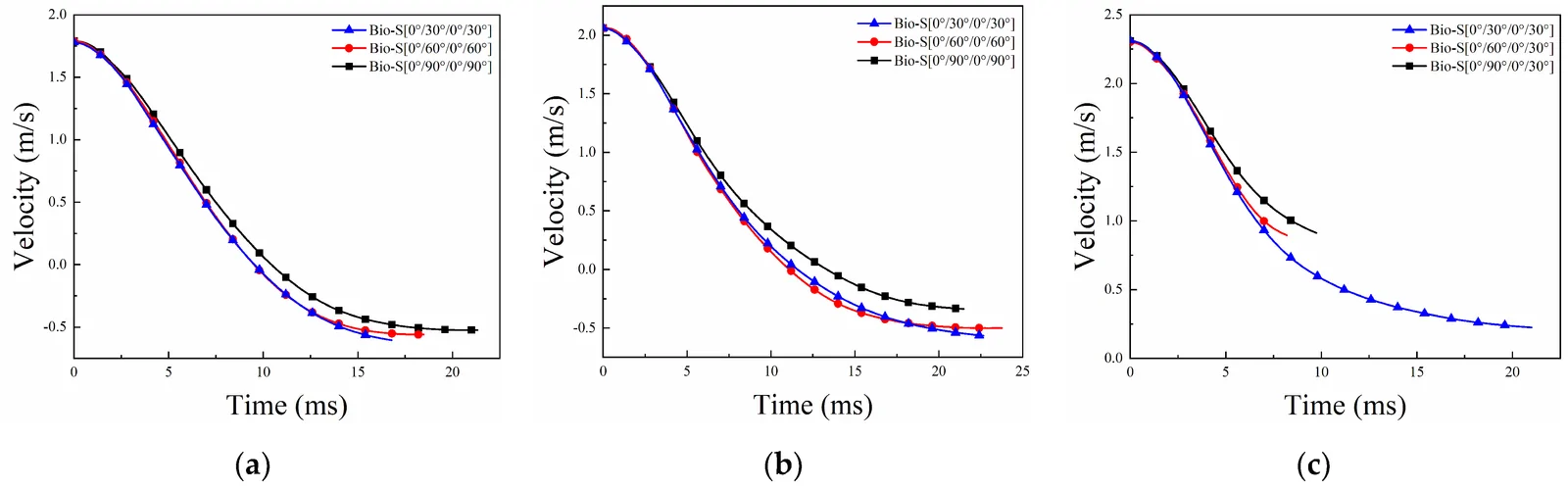
Velocity-time curves of the Bio-S composite plates with different angles of middle layer configurations under different impact energy levels: (**a**) 15 J; (**b**) 20 J; (**c**) 25 J.

**Figure 16:**
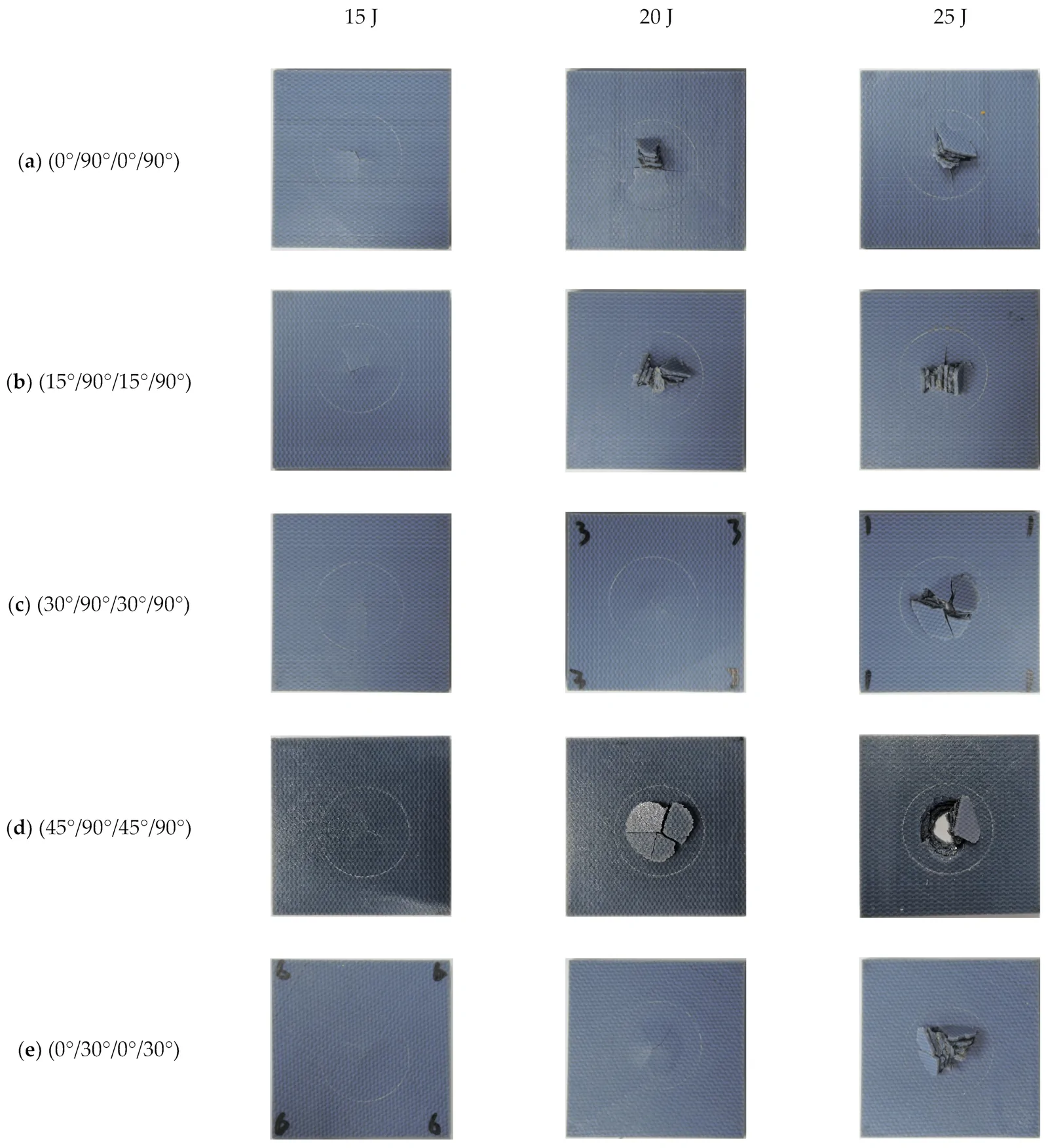
The damage morphologies of Bio-S with different layup configurations.

The force-displacement curves and velocity-time history curves of two group of samples obtained from the drop tower tests are shown in Figure 12, Figure 13, Figure 14 and Figure 15, while their damage morphologies are also presented in Figure 16. Table 3 lists EA, peak force, and ΔV for two group of samples. The first group, Bio-S with different ply angles, exhibited similar dynamic behaviors under the impact energy of 15 J. Evident elastic recovery was observed in the force-displacement curves as shown in Figure 12a, while the impact velocity dropped to zero and rebounded as shown in Figure 13a. This can be attributed to the impactor failing to go through the composite plate, whereas only a few cracks were observed on the backside of these composite plates (see deformations under the impact energy of 15 J in Figure 16a–d). From Figure 13b, only Bio-S with a layup of (45°/90°/45°/90°) had a residual velocity under the impact energy of 20 J, indicating that it could not withstand the crack propagation leading to crack holes of the impactor. In contrast, the velocities of other samples decreased to zero and rebounded, similar to those under the impact energy of 15 J, which also reflected in their fore-displacement curves with the elastic recovery, as shown in Figure 12b. Moreover, from Figure 16, for Bio-S with a layup of (30°/90°/30°/90°) plate, only a few cracks appeared and the impactor did not go through the plate, whereas Bio-S with other layup configurations suffered significant material damage and fracture near the impact location. It seemed that the Bio-S with layups of (30°/90°/30°/90°) and (45°/90°/45°/90°) exhibited the best and worst impact resistance under the impact energy of 20 J, respectively. As the impact energy was increased from 20 J to 25 J, however, the first group of specimens were completely going through, and there were large residual velocities in Figure 13c. As seen from Figure 16a–d, the most severe damage occurred in Bio-S with the layup of (45°/90°/45°/90°), with large pieces of material peeling away from the plate. Additionally, by comparing the residual velocities in Figure 13c, it can also be observed that Bio-S with the layup of (45°/90°/45°/90°) had the largest residual velocity, implying less EA, while Bio-S with the layup of (30°/90°/30°/90°) had the smallest residual velocity, showing the greatest resistance to crack propagation leading to crack holes in this group of specimens. Based on above analysis, it can be concluded that the ply angles of the first and third layers had a significant effect on the impact resistance of Bio-S composite plates. The highest impact resistance was achieved by Bio-S with the layup of (30°/90°/30°/90°), whereas the worst impact resistance was generated by Bio-S with the layup of (45°/90°/45°/90°).

Figure 14 and Figure 15 show the force-displacement curve and velocity-time history curve of the second group of Bio-S composite plates under low velocity impact, respectively, which also revealed that different ply angles of the second and fourth layers had a significant influence on the impact resistance. The second group of Bio-S composite plates could prevent the impactor from crack propagation leading to crack holes at the impact energy of 15 J and 20 J, but failed to withstand the impact energy of 25 J (see Figure 16a,c,e). However, under the impact energy of 25 J, the impact velocity can be attenuated from 2.31 m/s to 0.2 m/s by Bio-S with the layup of (0°/30°/0°/30°). Its residual velocity was very close to 0, implying that it almost resisted to the crack propagation leading to crack holes. Moreover, from Figure 14c, Bio-S with the layup of (0°/30°/0°/30°) exhibited a larger energy dissipation capacity owing to high load and large displacement, compared with Bio-S with layups of (0°/60°/0°/60°) and (0°/90°/0°/90°). From Table 3, both EA and ΔV of Bio-S with the layup of (0°/30°/0°/30°) had a significant increase compared with that of Bio-S with the layup of (0°/60°/0°/60°) that was same as (30°/90°/30°/90°) with the best impact resistance in the first group. Accordingly, it was considered that (0°/30°/0°/30°) was the optimal ply angle in the two groups of Bio-S composite plates, for the largest EA and ΔV under 25 J.

**Table 3:** EA, velocity change and peak force for Bio-S with the changing angles of middle layer configurations composite plate under different impact energy levels.

	EA (J)			ΔV (m/s)			Peak Force (N)		
Impact Energy	15 J	20 J	25 J	15 J	20 J	25 J	15 J	20 J	25 J
(0°/90°/0°/90°)	15.0	20.0	21.33	1.78	2.06	1.40	2214.8	2366.9	2237.3
(15°/90°/15°/90°)	15.0	20.0	22.23	1.78	2.06	1.51	2079.8	2176.6	2214.7
(30°/90°/30°/90°), (0°/60°/0°/60°)	15.0	20.0	22.21	1.78	2.06	1.52	2390.6	2559.1	2550.3
(45°/90°/45°/90°)	15.0	19.7	21.67	1.78	1.79	1.44	2302.4	2465.0	2685.0
(0°/30°/0°/30°)	15.0	20.0	23.67	1.78	2.06	1.52	2394.3	2494.0	2556.4

## 4. Crack Propagation Mode

### 4.1. Crack Propagation Mode

Figure 10 showed the damage morphologies of Bio-S, Bio-B, Bio-N, and MSP, where the photos of the bionic composite plates were taken by stereo optical microscope. Compared with MSP breaking into many pieces, Bio-S, Bio-B, and Bio-N maintained relative integrity, especially where there were only a few cracks that appeared in the back of Bio-S under 15 J impact energy. Furthermore, by observing and comparing the bionic composite plates and MSP after impact tests, the fracture surface of the bionic plates was uneven, which proved that they can provide more complex crack propagation mechanisms [31,32]. In contrast, it can be observed from Figure 10d that the fracture surface of MSP was smooth, which suggests that MSP broke instantly due to the rapid crack propagation during the impact. This relatively simple propagation mode of cracks led to less energy dissipation. Bio-S could maintain relative integrity after being impacted, which was crucial to resist multi impacts and protect the safety of internal equipment and person.

**Figure 17:**
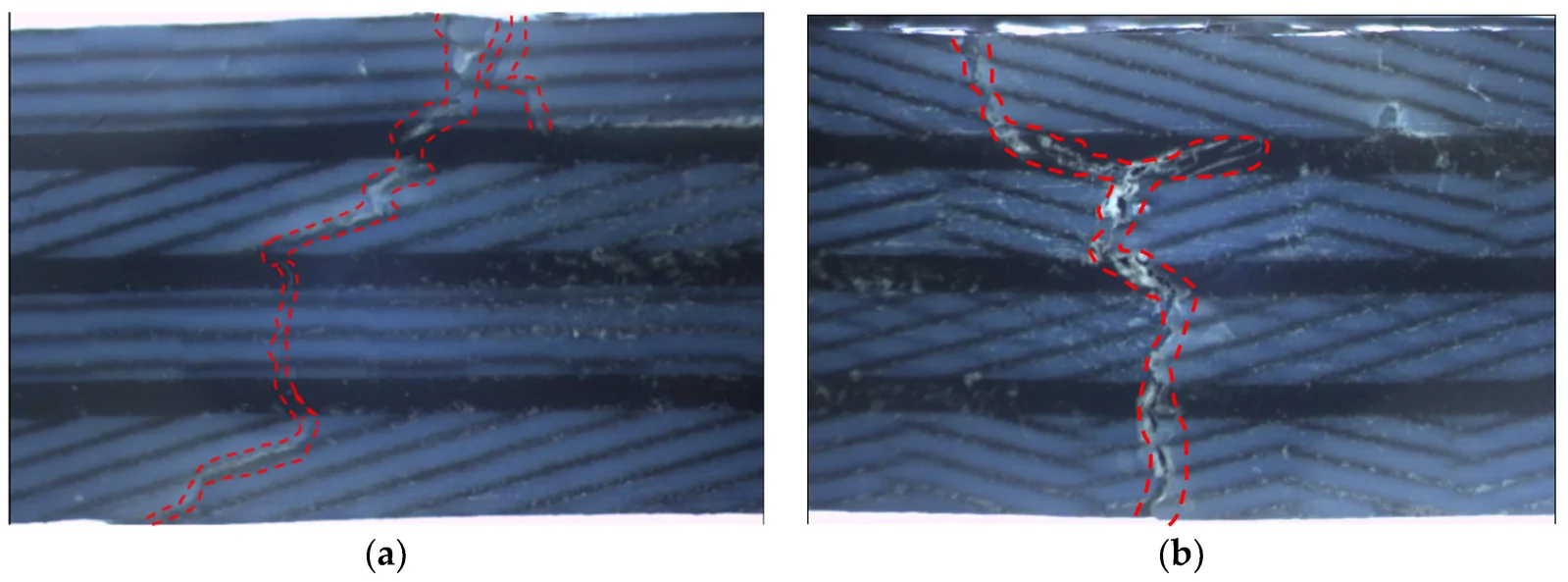
Crack propagation path in cross-section of Bio-S composite plate: (**a**) (30°/90°/30°/90°); (**b**) (0°/30°/0°/30°).

To observe the mode of crack propagation, the Bio-S composite plate in Figure 15 was cut along the centerline and photos of the section surface were taken by the stereo optical microscope. Figure 17a,b demonstrated the crack propagation for representative layups of (30°/90°/30°/90°) and (0°/30°/0°/30°), respectively. The propagation path of cracks revealed that the crack was initiated from the impact point location of Bio-S, and then deflected at the interface of the soft material between the first and second layers. It can also be observed from the highlight in Figure 17a that the crack traversed the stiff material and continued to propagate to the next layer with a ‘step-like’ propagation path. In addition, the highlight as shown in Figure 17b indicated that the crack would propagate along with the interface of the soft material for a certain distance and deflect with a large angle. The complicated mode, including crack initiation, propagation, and deflection, might be an important fracture toughness mechanism that Bio-S with layups of (30°/90°/30°/90°) and (0°/30°/0°/30°) exhibited excellent impact resistance compared with other stack configurations. Furthermore, the crack propagation paths observed at different layers and interfaces suggested that the damage was not concentrated at the impact area, but the different layers and interfaces of the structure could work together to resist the crack propagation leading to crack holes. Moreover, it can be concluded that the interfaces with stiff/soft combination that existed in the structure led to the crack deflecting or stopping during the impact, while dissipating more energy.

### 4.2. Crack Propagation Analysis

**Figure 18:**
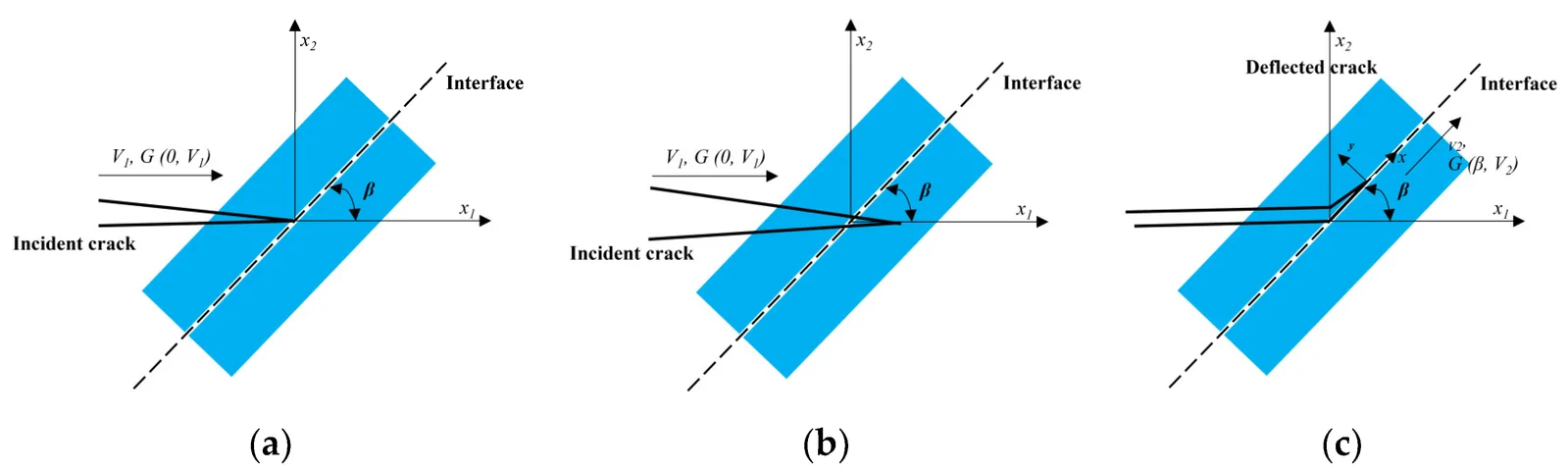
Schematic diagram of three crack growth modes: (**a**) Crack stops on the weak interface between two identical homogeneous solids; (**b**) crack goes through the weak interface between two identical homogeneous solids; (**c**) crack defects the weak interface between two identical homogeneous solids.

There were three modes when a crack reached the interface: first, the crack terminated at the interface (Figure 18a); second, the crack deflected into the interface (Figure 18b); and third, the crack traversed the interface, as shown in Figure 18c. In this paper, we focused on the latter two situations and developed an analytical model to discuss crack propagation.

Linear elastic fracture mechanics were adopted to define the conditions under which fracture occurs. The crack strength factor or energy release rate was less than the fracture toughness of the given material, and the structure would not fracture [37]. In the case of the structural interface, the deflection of the crack mean that the interface fractured and the crack propagated along with the interface; in the other case, the crack traversed the interface entering the matrix. The condition for crack deflection to enter the interface was [37]:

(1) $$\frac{G(\beta ,V_{2})}{G_{1}(V_{1})}\geq \frac{\Gamma_{Ιn}(V_{2})}{\Gamma_{Ma}(V_{1})}$$

where $G(\beta ,V_{2})$ and $G_{1}(V_{1})$ were the energy release rates of deflection crack and penetration crack, respectively. $\Gamma_{In}(V_{2})$ and $\Gamma_{Ma}(V_{1})$ were the fracture toughness of the interface layer and the matrix layer, respectively. $V_{1}$ and $V_{2}$ represented the incident and deflected crack speeds, respectively. β was defined as an interface angle for the interface direction with respect to the incident crack path.

The ratio of the energy release rate of the deflection crack on the left side of Equation (1) to the penetration crack was [35]:

(2) $$\frac{G\left(\beta ,V_{2}\right)}{G\left(V_{1}\right)}={\left(\frac{V_{2}}{V_{1}}\right)}^{2}\frac{D_{1}}{D_{2}}\frac{\alpha_{d2}k_{I}^{2}\left(V_{2}\right){\left(3\cos\frac{\beta }{2}+\cos\frac{3\beta }{2}\right)}^{2}+\alpha_{s2}k_{II}^{2}\left(V_{2}\right){\left(\sin\frac{\beta }{2}+\sin\frac{3\beta }{2}\right)}^{2}}{16\alpha_{d1}k_{I}^{2}\left(V_{1}\right)}$$

(3) $$\begin{array}{c} D=4\alpha_{s}\alpha_{d}-{\left(1+\alpha_{s}^{2}\right)}^{2},\alpha_{s}=\sqrt{1-{\left(\frac{V}{c_{s}}\right)}^{2}},\alpha_{d}=\sqrt{1-{\left(\frac{V}{c_{d}}\right)}^{2}}\\ c_{S}=\sqrt{\frac{\mu }{\rho }},c_{d}=\sqrt{\frac{\kappa +1}{\kappa -1}}c_{S},\kappa =\left\{\frac{3-4\upsilon \;(plane\;strain)}{\frac{3-\upsilon }{1+\upsilon }\;(plane\;stress)}\right. \end{array}$$

where *D* was a function related to the velocity, and *D*_1_ and *D*_2_ in the right-hand side of Equation (2) can be obtained by substituting $V_{1}$ and $V_{2}$ into *D*, respectively. $c_{s}$ and $c_{d}$ are the shear and expansion wave velocities of the matrix material, respectively. $k_{I}$ and $k_{II}$ are the universal function of the crack tip velocity and defined as [37]:

(4) $$k_{I}(V)=\frac{1-\frac{V}{c_{R}}}{\sqrt{1-\frac{V}{c_{d}}}}{\;k}_{II}(V)=\frac{1-\frac{V}{c_{R}}}{\sqrt{1-\frac{V}{c_{s}}}}$$

(5) $$c_{R}=\frac{0.862+1.14\upsilon }{1+\upsilon }c_{S}$$

where $c_{R}$ is the Rayleigh wave velocity of the material.

The above equations were applied to analyze the crack growth behavior of 3D-printed Bio-S with the stiff material of verowhite as the matrix and the soft material of tangoblackplus as the interface. The crack propagation speed in the matrix was $V_{1}=0.4c_{s}$ , and crack approached the structure interface at any angle, while the crack speed was $V_{2}=0$ if the crack was deflected [32]. For the stiff material verowhite, Young’s modulus, Poisson’s ratio, and density were E=2996 MPa , υ=0.3 , and $\rho =1175{\;\mathrm{kg}/m}^{3}$ from Table 1, respectively. The soft material properties of tangoblackplus can be obtained from reference [32]. Then $c_{s}$ , $c_{d}$ , and $c_{R}$ can be calculated with these values. The ratio on the right side of the Equation (1) was set to 1.5 [32] to determine the angle threshold of crack deflection or penetration. When the normalized energy release rate was greater than this value, the crack would traverse the interface, and vice versa, the crack was deflected into the interface [35].

**Figure 19:**
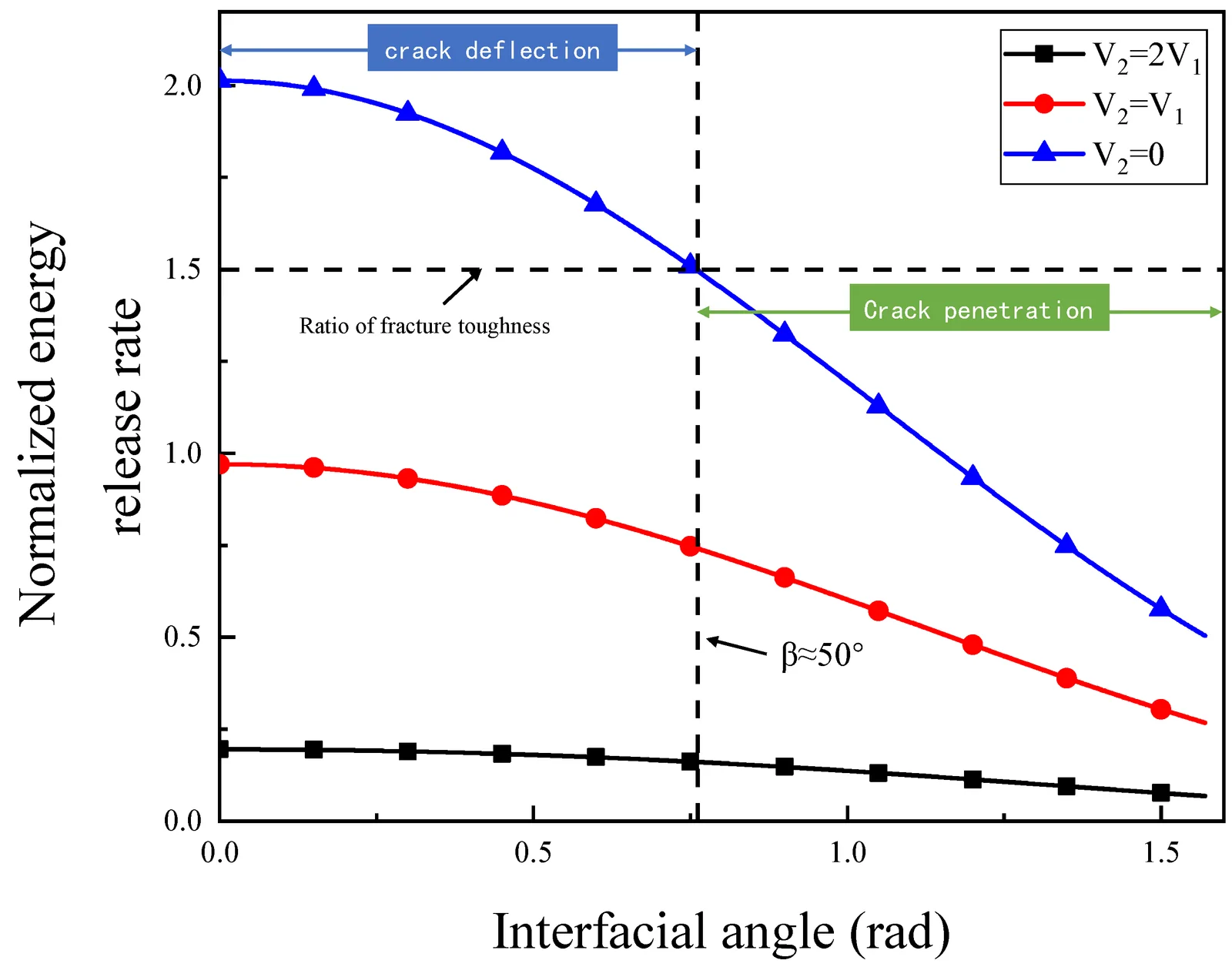
Schematic diagram of the normalized energy release rate interfacial angle curve.

Figure 19 showed the normalized energy release rate on the left of Equation (2) versus the interface angle β . If the crack was deflected, the ratio of fracture toughness on the right side of Equation (2) should be less than 1.5 and $V_{2}$ was equal to zero. Therefore, as shown in Figure 19, a horizontal line with a normalized energy release rate of 1.5 was drawn, and the intersection point with the curve corresponded to β about 0.87 rad (50°). Therefore, for the stiff/soft combination interface, according to Equation (2), it was considered that when β was less than 50°, the crack would deflect into the interface, whereas the crack would propagate through the interface for β > 50° [35]. Accordingly, the soft material determined the failure mode of the structure to a certain extent. The weaker the strength of the interface, the greater the range of deflection angle when the crack propagated, but the weaker interface strength affected the stiffness of the overall structure, leading to premature delamination failure of the structure. Therefore, it was necessary to obtain a balance for the filling amount of soft materials [32]. As shown in Figure 19, the threshold of β between crack deflection and penetration was close to 50°. When β was equal to 30°, the crack propagation tended to deflect, which was consistent with the optimal ply angle (0°/30°/0°/30°) of Bio-S.

## 5. Conclusions

In this work, a novel conch-inspired stiff–soft composite plate (Bio-S) was designed and compared with two previously developed stiff–soft composites, namely nacre-inspired (Bio-N) and beetle exoskeleton-inspired (Bio-B) plates, all of which were fabricated by multi-material 3D printing. The low-velocity impact damage behaviors and EA characteristics of these bionic composite plates were compared and assessed using drop tower tests. Then, the effect of the ply angle on the impact resistance of the Bio-S composite plates was studied. Furthermore, the crack propagation in Bio-S composite plates was analyzed and the underlying mechanisms were uncovered. The following conclusions were drawn from this paper:

(1) Through comparisons of EA, ΔV, and damage patterns of the designed bionic composite plates, Bio-S showed the best impact resistance, Bio-N showed the second best, while MSP and Bio-B showed the worst impact resistance;

(2) The ply angle had a significant influence on the impact resistance of the Bio-S composite plate. It was found that Bio-S with the layup of (0°/30°/0°/30°) could provide the highest impact resistance;

(3) The crack propagation mechanism and damage patterns with respect to interfacial strength and impact velocity revealed that the cracks in the Bio-S composite plate had a more complex mode and longer propagation path than MSP, accompanied by the presence of an enlarged contact area along with the microcrack formation.

The current work can provide new ideas for the design of novel armor for protective and defensive structures with excellent anti-impact performance by imitating natural strategies.

## Figures and Tables

**Table 1:** Mechanical properties parameters of verowhite.

	Density (kg/m^3^)	Elastic Modulus (MPa)	Poisson’s Ratio	Failure Stress (MPa)	Elongation
Value	1175	2996	0.3	55	30%

**Table 2:** EA, ΔV and peak force for each bionic composite plate under different impact energy levels.

	EA (J)			ΔV (m/s)			Peak Force (N)		
Impact Energy	15 J	20 J	25 J	15 J	20 J	25 J	15 J	20 J	25 J
Bio-S	15.0	20.0	21.33	1.78	2.06	1.40	2214.8	2366.9	2237.3
Bio-B [36]	9.27	10.7	11.74	0.68	0.66	0.62	2208.8	2166.4	2157.2
Bio-N [36]	15.0	19.9	20.1	1.78	1.87	1.27	1947.7	2158.2	2230.5
MSP [36]	13.6	13.6	11.7	1.23	0.89	0.62	7190.1	6891.1	6002.6

## Data Availability

Not applicable.
