# Correction: Wan et al. Performance of 3D-Printed Bionic Conch-Like Composite Plate under Low-Velocity Impact. *Materials* 2022, *15*, 5201

**DOI:** 10.3390/ma19183833

**Published:** 2026-09-09

**Authors:** Mincen Wan, Dayong Hu, Baoqing Pei

**Affiliations:** 1 Department of Aircraft Airworthiness Engineering, School of Transportation Science and Engineering, Beihang University, Beijing 100191, China; wanmincen@buaa.edu.cn; 2 Aircraft/Engine Integrated System Safety Beijing Key Laboratory, Beijing 100191, China; 3 School of Biological Science and Medical Engineering, Beihang University, Beijing 100191, China

## Missing Citation

In the original publication [1], Wang, J.; Hu, D.; Zhang, Z.; Pei, B.; Xu, R.; Wu, X. Anti-impact performance of bionic tortoiseshell-like composites. *Compos. Struct.* **2023**, *303*, 116315. https://doi.org/10.1016/j.compstruct.2022.116315 was not cited. The citation has now been inserted in the captions of Figures 5, 8–10 and content of Table 2, which should now read as follows:

**Figure 5.** Specimen of each structure: (**a**) Bio-S [0°/90°/0°/90°]; (**b**) Bio-B [0°/90°/180°], (**c**) Bio-N and (**d**) MSP. Reprinted with permission from Ref. [36]. Copyright 2022 Elsevier Ltd.

**Figure 8.** Comparison of force–displacement curves between Bio-S and the other three composite plates [36] under different impact energies: (**a**) 15 J; (**b**) 20 J; (**c**) 25 J.

**Figure 9.** Comparison of velocity-time curves between Bio-S and the other three composite plates [36] under different impact energy levels: (**a**) 15 J; (**b**) 20 J; (**c**) 25 J.

**Figure 10.** The damage patterns of Bio-S and the other composite plates. Reprinted with permission from Ref. [36]. Copyright 2022 Elsevier Ltd.

**Table 2:** EA, ΔV and peak force for each bionic composite plate under different impact energy levels.

	EA (J)			ΔV (m/s)			Peak Force (N)		
Impact Energy	15 J	20 J	25 J	15 J	20 J	25 J	15 J	20 J	25 J
Bio-S	15.0	20.0	21.33	1.78	2.06	1.40	2214.8	2366.9	2237.3
Bio-B [36]	9.27	10.7	11.74	0.68	0.66	0.62	2208.8	2166.4	2157.2
Bio-N [36]	15.0	19.9	20.1	1.78	1.87	1.27	1947.7	2158.2	2230.5
MSP [36]	13.6	13.6	11.7	1.23	0.89	0.62	7190.1	6891.1	6002.6

With this correction, the order of some references has been adjusted accordingly.

## Error in Figure

In the original publication [1], there was a mistake in Figures 10 and 13; the corrected Figure 10 and Figure 13 are shown below:

## Text Correction

There were some errors in the original publication [1]. Some text needs to be updated in the Abstract and Sections 1 and 5.

In the Abstract, the sentence “In this study, inspired by conch shell, beetle exoskeleton, and nacre, three different types of bionic composites plates were fabricated: Bio-S, Bio-B, and Bio-N, as well as an equivalent monolithic plate formed from the same stiff material designed and manufactured by additive manufacturing, respectively.” should be updated to “In this study, inspired by conch shell, a new type of bionic composite plate Bio-S was fabricated and compared with two previously investigated designs inspired by beetle exoskeleton (Bio-B) and nacre (Bio-N), as well as with an equivalent monolithic plate formed from the same stiff material designed and manufactured by additive manufacturing.”

In Section 1, Paragraph 4, the sentence “In addition, since nacre shell and beetle exoskeleton featured similar stiff-soft phase combination configurations to the conch shell, we also designed the bionic nacre-like and beetle exoskeleton composite plates to compare with the bionic conch-like composite plate.” should be updated to “In addition, since nacre shell and beetle exoskeleton featured similar stiff–soft phase combination configurations to the conch shell, previously developed bionic nacre-like and beetle exoskeleton composite plates [30] were also used to compare with the bionic conch-like composite plate.”

In Section 5. Paragraph 1, the sentence “In this work, three types of bionic stiff-soft phase composite plates with different architectures (Bio-S, Bio-B and Bio-N) were designed, inspired by biological armors and fabricated by multi-material 3D printing technique.” should be updated to “In this work, a novel conch-inspired stiff–soft composite plate (Bio-S) was designed and compared with two previously developed stiff–soft composites, namely nacre-inspired (Bio-N) and beetle exoskeleton-inspired (Bio-B) plates, all of which were fabricated by multi-material 3D printing.”

The authors state that the scientific conclusions are unaffected. This correction was approved by the Academic Editor. The original publication has also been updated.

## Figures and Tables

**Figure 10:**
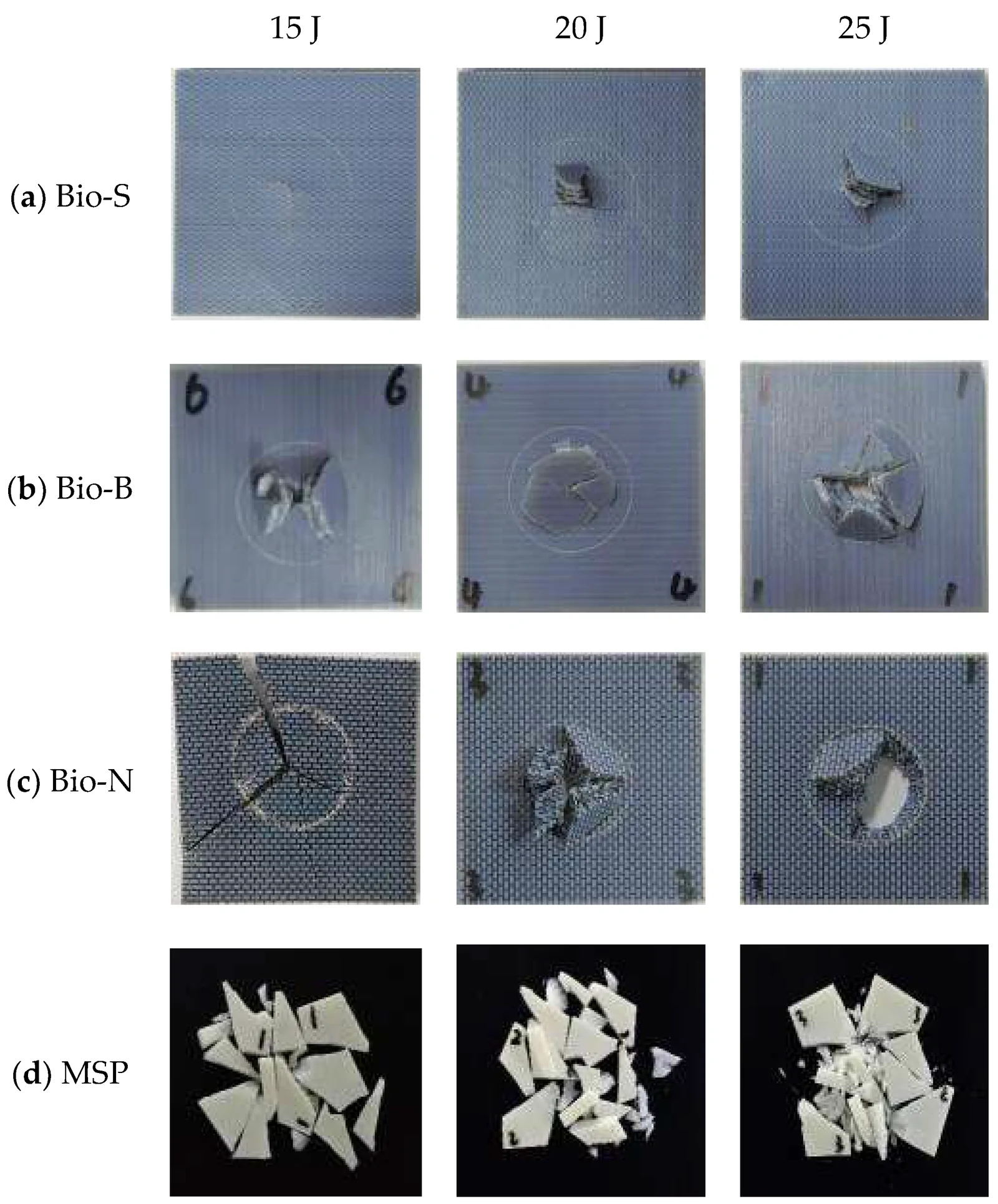
The damage patterns of Bio-S and the other composite plates. Reprinted with permission from Ref. [36]. Copyright 2022 Elsevier Ltd.

**Figure 13:**
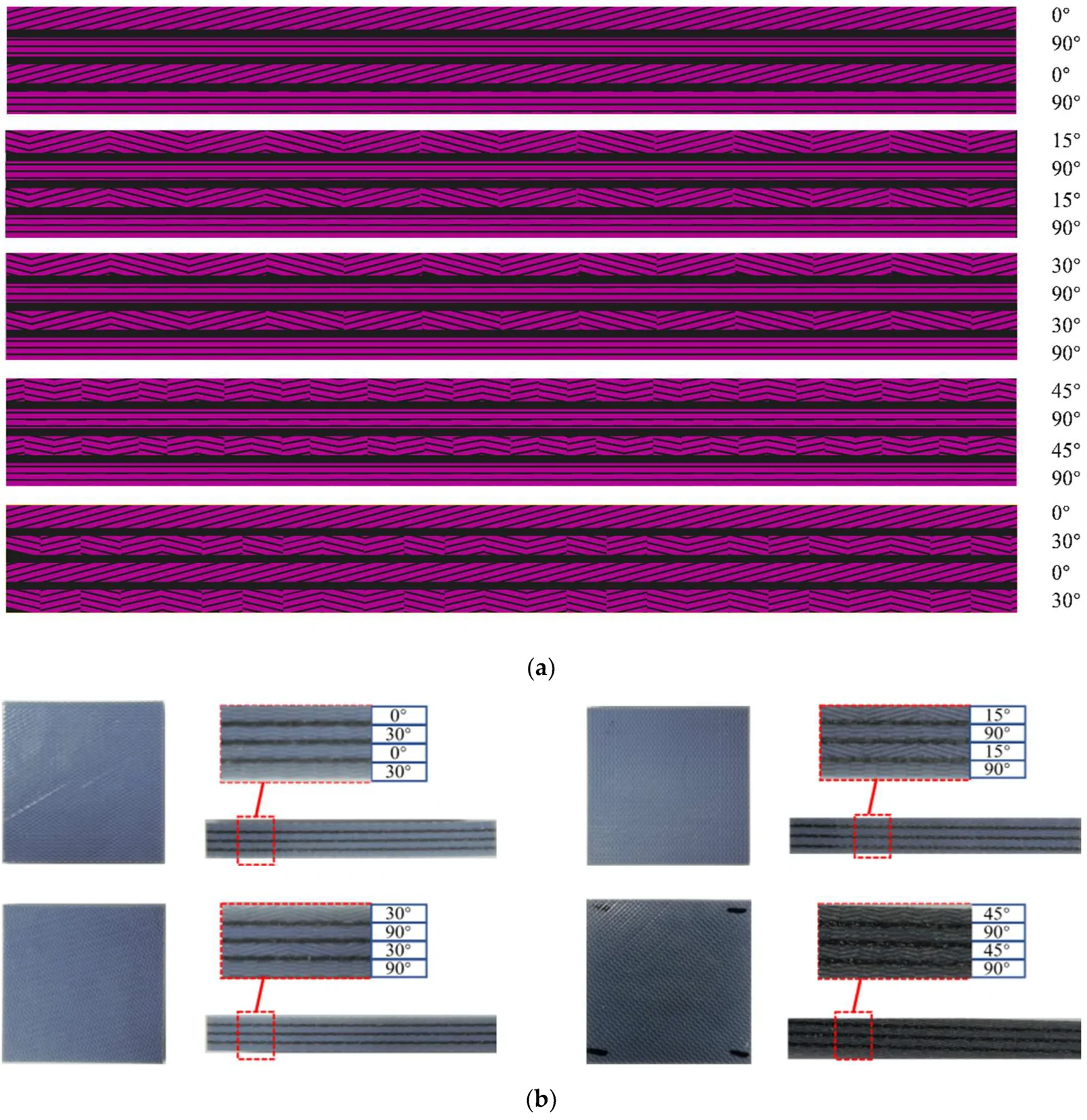
(**a**) Schematic diagram of different layers of Bio-S; (**b**) the corresponding test specimens.
